# Anchor Point Selection: Scale Alignment Based on an Inequality Criterion

**DOI:** 10.1177/0146621621990743

**Published:** 2021-02-25

**Authors:** Carolin Strobl, Julia Kopf, Lucas Kohler, Timo von Oertzen, Achim Zeileis

**Affiliations:** 1 Universität Zürich, Switzerland; 2 Universität der Bundeswehr München, Germany; 3 Universität Innsbruck, Austria

**Keywords:** differential item functioning (DIF), item bias, anchor items, item clusters

## Abstract

For detecting differential item functioning (DIF) between two or more groups of test takers in the Rasch model, their item parameters need to be placed on the same scale. Typically this is done by means of choosing a set of so-called anchor items based on statistical tests or heuristics. Here the authors suggest an alternative strategy: By means of an inequality criterion from economics, the Gini Index, the item parameters are shifted to an optimal position where the item parameter estimates of the groups best overlap. Several toy examples, extensive simulation studies, and two empirical application examples are presented to illustrate the properties of the Gini Index as an anchor point selection criterion and compare its properties to those of the criterion used in the alignment approach of Asparouhov and Muthén. In particular, the authors show that—in addition to the globally optimal position for the anchor point—the criterion plot contains valuable additional information and may help discover unaccounted DIF-inducing multidimensionality. They further provide mathematical results that enable an efficient sparse grid optimization and make it feasible to extend the approach, for example, to multiple group scenarios.

## Introduction

One of the major advantages of probabilistic test theory is that its assumptions are empirically testable. With regard to test fairness, a crucial step in test validation is to identify items that exhibit differential item functioning (DIF) for different groups of test takers. DIF items can lead to unfair test decisions and threaten the validity of the test (cf., e.g., Cohen et al., 1996; Magis & De Boeck, 2011) as well as its acceptance from the side of the test takers and policy makers. Once DIF items are identified, they can be improved or excluded from the final test form (cf., e.g., Westers & Kelderman, 1992). But, to identify them, first the item parameters of the groups need to be placed on the same scale in a way that allows to compare the individual item parameters between the groups. This is usually done by choosing a set of so-called anchor items.

A large body of literature has been discussing and investigating different strategies for selecting these anchor items for DIF testing, particularly for the Rasch model (see, e.g., Teresi & Jones, 2016, for a recent and broad overview on anchoring and DIF testing techniques). Questions that are being addressed in this literature—but have not all been answered satisfactorily yet—include the choice of the number of items (also termed anchor length) as well as different strategies to select those items. Only a few exemplary anchoring methods, namely those that will be later used as comparison methods in the simulation studies, will be seen in detail in the following. These methods have been selected to represent different approaches for anchor item selection, which are either widely used or have shown high performance in previous studies.

### Exemplary Anchor Item Selection Methods

The first example that will be treated in a little more detail here is the anchor method suggested by Woods (2009), which is classified as the “constant all other” method by the taxonomy of Kopf et al. (2015b). It is an anchor of fixed length, which is selected based on the “all other” strategy: In the initial step, each item is tested for DIF using all other items together as the preliminary anchor. For this and the following method, will be fixed the anchor length to four items in the following simulation studies.^1^ The four items corresponding to the lowest ranks of the absolute DIF statistics from the initial step are then chosen as the final set of anchor items. This method represents a commonly used and simple approach. However, by using all remaining items as the anchor in the initial step, this method (just like the similarly common “equal mean” approach, e.g., Magis & De Boeck, 2011) assumes that DIF is balanced and cancels out over the items. If, however, DIF is not balanced, this strategy has been shown to exhibit a severely increased false alarm rate (Kopf et al., 2015a, 2015b).

The second example is the “constant four mean *p*-value threshold” (later abbreviated as “constant four MPT”) anchor method suggested by Kopf et al. (2015a). Its selection of four anchor items is based on the number of *p* values that exceed a threshold *p* value determined from preliminary DIF tests for every item with every other item (one at a time) as a single anchor item (for a more detailed description, see Kopf et al., 2015a). This method, together with the one described next, has been shown to be one of the two top performing methods in the extensive comparison study of Kopf et al. (2015a) and thus serves as a strong competitor here.

Another example is the “iterative forward mean test statistic threshold” (later abbreviated as “iterative forward”) anchor method suggested by Kopf et al. (2015b). This method iteratively selects an anchor of variable length in a step-by-step procedure. The order in which new items are included in the anchor is determined by the mean test statistic threshold criterion. The rationale behind this criterion is that those DIF tests where the anchor is truly DIF free should display the least absolute mean test statistics. Note, however, that the definition of the threshold depends on the assumption that the majority of the items are DIF free. More details are provided in Kopf et al. (2015a).

### Scale Indeterminacy and Anchoring for DIF Detection

Going back one step in the reasoning, the fact that an anchor has to be chosen in the first place is due to the scale indeterminacy of the Rasch model (see, e.g., Fischer & Molenaar, 1995). Anchoring solves this indeterminacy in a way that allows the item parameters of the groups to be compared to detect DIF. This is usually achieved by placing the same restriction on the item parameters in both groups (as formalized, e.g., by Eggen & Verhelst, 2006; Glas & Verhelst, 1995) to define a common scale. The reason why anchoring is needed is that it is necessary to separate DIF from true differences in the mean abilities between the groups (often termed impact) by means of somehow conditioning on an estimate of the ability (DeMars, 2010; Lord, 1980; Van der Flier et al., 1984). From a practical point of view, it cannot be known in advance which items are the ones that have DIF and which are the ones that do not. Ideally, those items that end up being selected into the anchor should be DIF free, because otherwise the false alarm rate of the DIF tests increases, as shown, for example, by Wang et al. (2012), but in practice there is no way to check in an empirical setting whether the anchor selection worked properly.

In the DIF literature, several assumptions and notions can be found about DIF and DIF detection that are not always made very explicit. For example, anchoring methods may only work properly if DIF is balanced (as discussed for the “all other” and “equal mean” strategies above), or assume implicitly or explicitly that the majority of items is DIF free (as discussed explicitly by Kopf et al., 2015a), but implicitly underlying several other anchor methods as well). Note that in a real data analysis (as opposed to a simulation study, where the DIF structure is known) neither assumption can be approved or empirically tested a priori, so that users should critically assess whether these assumptions are plausible in their case and how grave the consequences of a deviation from the assumption would be (such as a severely inflated false alarm rate for the equal mean and constant “all other” methods in case the DIF is not balanced). It will be further discussed below that the assumption that the majority of items is DIF free may seem particularly plausible for many tests, because we know how much time and effort the content experts have spent on putting the items together. However, from a methodological point of view, it may restrict the theoretical thinking about the general concepts of anchoring and DIF, and is also critically discussed by Bechger and Maris (2015) and Pohl et al. (2017).

When DIF is considered from the point of view of multidimensionality (e.g., Ackerman, 1992; Roussos & Stout, 1996), it becomes clear that the assumption that the majority of items is DIF free corresponds to the assumption that the majority of items measure the primary dimension of interest, and nothing else. In this framework, it has been shown that DIF can result from secondary dimensions, for which the distributions of two groups of test takers differ (for details see Ackerman, 1992; Roussos & Stout, 1996), and which some of the items measure in addition to the primary dimension. If only few individual items measure secondary dimensions, this is perfectly in line with the assumption that the majority of items measures the primary dimension and should be considered DIF free. If we think of scenarios, however, where clusters of items measure the same secondary dimensions (including scenarios where the primary dimension no longer provides the majority of items, as will be illustrated below), it would be helpful to be able to detect this kind of pattern.

### Outlook on the Contents of This Manuscript

This manuscript follows an approach that is different from the “traditional” anchor item selection methods described above. Rather than assessing only certain combinations of anchor items, the idea of this approach is to align the two scales by optimizing an objective function, which captures the discrepancy between the scales along a continuum of potential anchor points. Specifically, the authors propose to maximize the inequality of item-wise absolute distances—captured by the so-called Gini Index, an inequality criterion from economics—to find an anchor point where very few items (if any) exhibit DIF, while most other items do not. This approach has turned out to be closely related to—but was developed independently of—the alignment method by Asparouhov and Muthén (2014) and Muthén and Asparouhov (2014), who employ the so-called component loss function as the criterion for selecting an optimal anchor point.

Although the motivation of both approaches was to select anchor points without using anchor items, it turns out somewhat surprisingly that optimal anchor points may in fact correspond to single anchor items. This is shown mathematically for both criteria, the Gini Index and the component loss function, for the case of a Rasch model based on conditional maximum likelihood (CML) estimation in two groups. Note that neither Asparouhov and Muthén’s work nor the previous version of our own manuscript (Strobl et al., 2018) had pointed out this property, which greatly facilitates searching for the optimal anchor point solution.

Despite this simple optimal solution, in the following the authors will first explain the general idea of shifting the scales along a continuum of potential anchor points, which establishes more broadly the idea of an optimal point where the item parameters best interlock. Moreover, in addition to the globally optimal solution, the pattern of potential local optima can be particularly informative, as will be shown in several illustrations.

Now the authors will first introduce a little notation and review the fundamentals of anchoring. Then the new approach for finding anchor points will be introduced. Its usefulness will be illustrated by means of illustrative toy examples, an extensive simulation study as well as two application examples, where DIF between female and male test takers will be investigated. Due to space constraints, many illustrations and results have been moved to online appendices, to which the reader will be referred to in due course. In particular, Online Appendix F provides the mathematical derivation of the possible locations of optima for both criteria. In the discussion, the authors will also point out the possibility to extend their approach to settings with more parameters and multiple groups.

## Anchoring Revisited

Due to its scale indeterminacy, that is, the fact that the latent scale has no natural origin, a restriction is necessary for estimating the item parameters in the Rasch model. Commonly used restrictions are setting (arbitrarily) the first item parameter or the sum of all item parameters to zero (Eggen & Verhelst, 2006; Glas & Verhelst, 1995). When the aim is to compare the item parameters between two groups, the item parameters are first estimated separately. In the following, these initial item parameter estimates will be termed ${\tilde{\beta }}_{j}^{\left(g\right)}$ for group g and item j. Since any linear restriction can easily be obtained from any other, it does not matter which particular restriction is applied in this first step. However, the restriction used for DIF detection is a critical choice, as illustrated in Online Appendix A.

### The Choice of the Restriction

Considering the choice of a suitable restriction for comparing the item parameters of two groups, a variety of strategies has been suggested to choose a set of suitable anchor items. The sum of the item parameters of this set of anchor items is usually set to zero in both groups as the new restriction. In the following, the authors will introduce and explain some notation for describing the process of anchoring mathematically.

The authors start off with the initial item parameter estimates for each group, ${\tilde{\beta }}_{j}^{\left(g\right)}$. In the following, they will employ the CML approach for estimating the item parameters, but the general principle outlined here applies to any kind of item parameter estimates.^2^

In the notation for describing the process of anchoring, let A denote the set of anchor items and |A| its cardinality, i.e., the number of anchor items in this set. The restriction that the sum of the anchor item parameters should be zero in group g can then be expressed as $\sum_{j\in A}{\hat{\beta }}_{j}^{\left(g\right)}\overset{!}{=}0$. The final item parameter estimates ${\hat{\beta }}_{j}^{\left(g\right)}$ can be derived from the initial estimates ${\tilde{\beta }}_{j}^{\left(g\right)}$ by means of shifting all item parameters by

$${\hat{\beta }}_{j}^{\left(g\right)}={\tilde{\beta }}_{j}^{\left(g\right)}-\frac{\sum_{j\in A}{\tilde{\beta }}_{j}^{\left(g\right)}}{\left|A\right|}.$$

This shift ensures that the sum of the anchor item parameters is zero in each group. Of course, all other item parameters are also shifted by the same amount, so that the overall pattern of the item parameters in each group is not altered, but moved as a whole to a position where it can best be compared with the pattern of item parameters in the other group.

More abstractly speaking, the process of anchoring corresponds to shifting all item parameters by a constant $c^{\left(g\right)}$

$${\hat{\beta }}_{j}^{\left(g\right)}={\tilde{\beta }}_{j}^{\left(g\right)}-c^{\left(g\right)},$$

where in all traditional anchoring approaches $c^{\left(g\right)}=c^{\left(g\right)}(A)=\frac{\sum_{j\in A}{\hat{\beta }}_{j}^{\left(g\right)}}{\left|A\right|}$ depends on the choice of the anchor set A and can take all values that result from the different combinations of anchor items that are being in- or excluded in A.

Conceptually, it is possible to uncouple the shift of the item parameters from a certain choice of anchor items. This can be accomplished by means of searching over an interval $\left[c_{\min},c_{\max}\right]$ of values for $c^{\left(g\right)}$, including values that do not result from any specific combination of anchor items.

Without loss of generality, rather than shifting the item parameters of both groups, the item parameters of the first group are left at their initial estimates

$${\hat{\beta }}_{j}^{\left(g_{1}\right)}={\tilde{\beta }}_{j}^{\left(g_{1}\right)}\;\mathrm{with}\;c^{\left(g_{1}\right)}=0,$$

where any arbitrary restriction can be used for the initial estimates ${\tilde{\beta }}_{j}^{\left(g_{1}\right)}$. The item parameters of the second group are then “moved past” the item parameters of the first group by means of shifting them by a constant c:

$${\hat{\beta }}_{j}^{\left(g_{2}\right)}={\tilde{\beta }}_{j}^{\left(g_{2}\right)}-c^{\left(g_{2}\right)}\;\mathrm{with}\;c^{\left(g_{2}\right)}=c.$$

For the boundaries of the interval $\left[c_{\min},c_{\max}\right]$, values can then be used such that the item parameter ranges of both groups are safely overlapping:

$$\left[c_{\min},c_{\max}\right]=\left[\min\left({\tilde{\beta }}^{\left(g_{1}\right)}\right)-\max\left({\tilde{\beta }}^{\left(g_{2}\right)}\right),\max\left({\tilde{\beta }}^{\left(g_{1}\right)}\right)-\min\left({\tilde{\beta }}^{\left(g_{2}\right)}\right)\right].$$

This means that the item parameters of the second group are moved fully past the item parameters of the first group, starting where the lowest item of the first group interlocks with the highest item of the second and moving on until the highest item of the first group interlocks with the lowest item of the second group. Below we will see in detail how the shift constant c can be selected based on the data by searching over this interval (or over a more sparse grid, as derived in Online Appendix F).

For the final DIF test, we will then look at a test statistic based on the difference between the final item parameter estimates of the two groups on the shifted scale: ${\hat{\beta }}_{j}^{\left(g_{1}\right)}-{\hat{\beta }}_{j}^{\left(g_{2}\right)}={\tilde{\beta }}_{j}^{\left(g_{1}\right)}-{\tilde{\beta }}_{j}^{\left(g_{2}\right)}-c$. Note that this comparison depends on the choice of c, which will be selected in a suitable way, but not on the choice of the initial restrictions, because the selection of c will make up for any shift in the ${\tilde{\beta }}^{g}$.

A common choice of such a test statistic for the final DIF test is that of the item-wise Wald test

$$t_{j}=\frac{{\hat{\beta }}_{j}^{\left(g_{1}\right)}-{\hat{\beta }}_{j}^{\left(g_{2}\right)}}{\hat{s}e_{j}}=\frac{{\tilde{\beta }}_{j}^{\left(g_{1}\right)}-{\tilde{\beta }}_{j}^{\left(g_{2}\right)}-c}{\hat{s}e_{j}},$$

with ${\hat{\mathrm{se}}}_{j}=\sqrt{\tilde{\mathrm{Var}}{\left({\tilde{\beta }}^{\left(g_{1}\right)}\right)}_{j,j}+\tilde{\mathrm{Var}}{\left({\tilde{\beta }}^{\left(g_{2}\right)}\right)}_{j,j}}$. Note that the item-wise Wald test is applied to the conditional maximum likelihood estimates in the following (like in Glas & Verhelst, 1995; Kopf et al., 2015a, 2015b).

When reconsidering the idea of moving the item parameters of the second group past those of the first group, for us as human beings it is straightforward that some positions are smarter than others, but the crucial question is: Can we find an objective criterion to make this decision for us automatically—both to avoid subjectiveness in our decision and to make it computationally feasible?

At first sight it may seem like c could be optimized directly with respect to a test statistic like that of the Wald test displayed above, or with respect to some kind of norm ||d(c)|| of the vector $d(c)=(d_{1}(c),\ldots ,d_{m}(c){)}^{T}$ of the item-wise absolute distances on the shifted scale

$$d_{j}(c)=\left|{\hat{\beta }}_{j}^{\left(g_{1}\right)}-{\hat{\beta }}_{j}^{\left(g_{2}\right)}\right|=\left|{\tilde{\beta }}_{j}^{\left(g_{1}\right)}-{\tilde{\beta }}_{j}^{\left(g_{2}\right)}-c\right|.$$

Measures based on these distances could capture what could be called the *overall amount* of DIF, for example, by using the sum of squared (Euclidean) or absolute (Cityblock) distances (corresponding to the L2 or L1 norm) as the criterion. However, a norm-based criterion could become large both if there are many small differences or a few large differences in the vector d(c). For DIF detection and interpretation, however, these would have very different meanings.

It will be shown in the next section that DIF detection can better be achieved by applying a measure of *inequality* instead of a measure of the *overall amount* of DIF to d(c) by using, for example, the popular Gini Index as the criterion.

### The Gini Index

As an objective criterion for automatically selecting anchor points, the authors suggest to use the Gini Index (Gini, 1912/1955). The Gini Index is a popular inequality measure, which is usually employed for assessing the distribution of wealth or income between the members of a society. It takes high values if, for example, a small minority of persons has a lot of wealth while the vast majority has very little. It is therefore used to compare different countries with respect to their distribution of wealth or income (e.g., Central Intelligence Agency, 2017).

The authors will now show how the Gini Index can also be used as a means for selecting anchor points. This is most easily imagined when the majority of items displays no DIF. Then at the optimal anchor point, where the scales for the two groups are aligned as well as possible, most items will interlock (i.e., they will lie on top of or very close to each other for the two groups), while a minority of items will differ for the two groups and show DIF. So while initially the Gini Index was used to indicate whether a minority of *persons* has a lot of *wealth* and the majority has very little, it will be used here to find solutions where a minority of *items* has a lot of *DIF* (i.e., large absolute differences in their item parameter estimates between the groups) while the majority has very little or no DIF (i.e., small or no absolute differences in their item parameter estimates between the groups).

The Gini Index can be computed as

$$\mathrm{GI}(c)=\frac{2\cdot \sum_{j=1}^{m}r_{j}(c)\cdot d_{j}(c)}{m\cdot \sum_{j=1}^{m}d_{j}(c)}-\frac{m+1}{m},$$

where $r_{j}(c)$ is the rank of the absolute item-wise distance $d_{j}(c)$ for item j, with j=1,…,m.

The optimal anchor point based on the Gini Index then corresponds to

$$c_{\mathrm{maxGI}}=\underset{c\epsilon \left[c_{\min},c_{\max}\right]}{\arg\max}\mathrm{GI}(c).$$

The Gini Index can take values between 0 and close to 1. The value zero corresponds to perfect equality, that is, all items having the same absolute item-wise distances, in which case they can be shifted such that the two groups are perfectly aligned and no item displays DIF.^3^ Values close to one, on the other hand, correspond to perfect inequality, where one item has all the DIF (i.e., a high absolute item-wise distance) while all other items have no DIF at all. In this case, the Gini Index reaches its maximum possible value of $1-\frac{1}{m}$. For example, if one out of 10 items had DIF and the remaining nine items would have no DIF at all, its maximum would be $1-\frac{1}{m}=1-\frac{1}{10}=0.9$.

Note that the value of the Gini Index in this example depends only on the number of items, not on the absolute amount of DIF. This property of the Gini Index, that it is independent of the absolute amount of wealth (i.e., it does not measure the absolute effect size of DIF, but the strength of the inequality of the distribution of DIF among the items), is further illustrated below.

The authors will show how selecting c according to the Gini Index leads to shifts between the two groups that makes their item parameters well comparable. This approach can serve as the basis for any kind of graphical display as well as for formal DIF tests. We will also see that the Gini Index is able to detect multiple clusters of items in the case of unaccounted DIF-inducing multidimensionality.

### The Component Loss Function Criterion Used by Asparouhov and Muthén

Asparouhov and Muthén (2014) and Muthén and Asparouhov (2014), coming from a factor analysis background, describe that their alignment method was first motivated by the task to estimate group-specific factor means and variances for many groups at a time, which the authors explain is not feasible by means of modification indices (Asparouhov & Muthén, 2014). As a by-product, the result can also be used for measurement invariance analysis, that is, for detecting DIF.

Asparouhov and Muthén (2014) introduce their approach in a factor analysis notation and framework, but Muthén and Asparouhov (2014) show how it translates to the case of a two-parameter logistic item response theory (IRT) model. Here the authors will refer to the so-called simplicity function and component loss function (CLF) used by Asparouhov and Muthén (2014) and Muthén and Asparouhov (2014), which will be explained in detail below. They will adopt the CLF as an alternative criterion for selecting optimal anchor points in their framework based on CML estimation for the Rasch model. It will be illustrated below that, compared with the Gini Index, it has similar properties in some but distinct properties in other DIF settings. Moreover, it is shown mathematically in Online Appendix F that both the Gini and the CLF Criterion can only find optima in single items in this particular framework, which makes the selection computationally much more feasible.

Note that the application of this criterion in this framework, based on CML estimation for the Rasch model, means that certain properties of Asparouhov and Muthén’s approach, which was originally described for a two-parameter model and for optimizing means and variances, may not carry over (in particular any effects of DIF affecting group variances). Yet, concentrating on this simple case allows us to concentrate on some fundamental properties of the criteria and compare the results to the extensive existing literature on DIF testing in the Rasch model.

The authors will now translate the simplicity function and CLF used by Asparouhov and Muthén into their notation. At the core of both the authors' and Asparouhov and Muthén’s reasoning is the idea to find a criterion that can be optimized such that “there are a few large noninvariant measurement parameters and many approximately invariant measurement parameters rather than many medium-sized noninvariant measurement parameters” (Asparouhov & Muthén, 2014, p. 497). This aim corresponds exactly to the authors' initial idea when using the Gini Index: to find solutions where a minority of items has a lot of DIF while the majority has very little or no DIF. Asparouhov and Muthén (2014) motivate their approach by earlier suggestions for criteria for finding simple structure solutions in factor rotation. The authors will show in the following that inequality criteria behave very similarly and argue that it may be fruitful to further explore the mathematical and philosophical similarities and specifics of the criteria used here, as well as potential further criteria from both research areas.

Because Asparouhov and Muthén (2014) consider a two-parameter model, their simplicity function *F* (Equation 9, Asparouhov & Muthén, 2014, p. 497) consists of a sum over both types of parameters (as well as over multiple groups). In the simpler case of the Rasch model with only one parameter (and the case of two groups), in our notation with $d_{j}(c)$ again representing the absolute distances in the difficulty parameters of item j between the two groups at point c in the search grid, the simplicity function becomes

$$F(c)=\sum_{j=1}^{m}\;f_{\epsilon }\left(d_{j}(c)\right),$$

with $f_{\epsilon }(d_{j}(c))$ denoting the CLF.

Asparouhov and Muthén use the particular form

$$f_{\epsilon }\left(d_{j}(c)\right)=\sqrt{\sqrt{d_{j}{\left(c\right)}^{2}+\epsilon }}$$

for the CLF, where the small positive constant ϵ is only added to ensure continuous differentiability for making the optimization easier. Since a grid-based rather than a gradient-based search is used here for the optimal value of c, it is not necessary to add ϵ. Therefore, the simplified CLF

$$f\left(d_{j}(c)\right)=\sqrt{\sqrt{d_{j}{\left(c\right)}^{2}}}=\sqrt{d_{j}(c)}$$

is used throughout this manuscript for mathematical coherence and simplicity. This has been checked that this makes no notable difference for any of the empirical results. The use of this particular form of the CLF is motivated by Asparouhov and Muthén (2014) and Muthén and Asparouhov (2014) through its being a “good choice” among component loss functions, which are being used in exploratory factor analysis to find rotations to simple structure solutions. The optimal anchor point based on the CLF then corresponds to

$$c_{\mathrm{maxCLF}}=\underset{c\in \left[c_{\min},c_{\max}\right]}{\arg\max}-\sum_{j=1}^{m}\;f\left(d_{j}(c)\right).$$

Note that throughout the main part of this manuscript the authors maximize and display $-\sum_{j=1}^{m}\;f(d_{j}(c))$ (rather than minimizing $\sum_{j=1}^{m}\;f(d_{j}(c))$ like Asparouhov and Muthén) and refer to this as the CLF Criterion in the following. They do this so that, both for the Gini Index and the CLF Criterion, larger values correspond to more inequal distributions of DIF, and maxima can be interpreted as optimal solutions. The shape of both criteria is illustrated in Online Appendix B.

Another difference to the original approach by Asparouhov and Muthén is that they concentrate on the use of the CLF in an automated process for the detection of a single global optimum, viewing multiple local optima as more of a nuisance, while the authors argue that those situations where distinct local optima occurr in addition to the global optimum are worth exploration by content experts. This is why, in addition to aggregated results, the authors will later display plots of the criterion values over the entire search interval for both the Gini Index and the CLF Criterion.

We would also like to point out that related notions of additional restrictions to be placed on the item parameter estimates to enhance comparability (e.g., Glas & Verhelst, 1995; von Davier & von Davier, 2007) and more or less algorithmic approaches for deciding which item parameters should be allowed to differ between groups (e.g., Glas & Jehangir, 2014; Oliveri & von Davier, 2014; Yamamoto et al., 2013) have been suggested and used for a long time by other authors. Pokropek et al. (2020) also point out the connection to the literature on linking and equating. However, the approach of Asparouhov and Muthén is most closely related to the one presented here. Therefore, in the following we will concentrate on investigating the properties of the Gini Index and the CLF Criterion for selecting anchor points in greater detail.

## Illustration of the Properties of Gini Index and CLF Criterion

The remainder of this article, together with the Online Appendices C through E, provides several illustrations of the properties of the new anchor point selection approach based on the Gini Index under a variety of settings. First, the authors will show by means of a few toy examples how both the Gini Index and the CLF Criterion detect the optimal shift value for aligning the item parameters in both groups. Second, the authors will present results from an extensive simulation study, where these methods are compared with each other as well as to existing anchoring approaches from the literature. By means of additional illustrations, the authors will show that the Gini Index shows a behavior that reflects their earlier considerations and that its criterion plot reflects additional information about the underlying pattern of the items particularly well. In Online Appendix E the authors will illustrate the practical usage of the approach by means of two empirical examples.

First, a very simple DIF pattern will be considered to highlight the properties of the two criteria we can use for anchor point selection. In this first example 10 items have been simulated, of which one item (Item 4) has been simulated with DIF. The authors will first present an illustration for the true values of the item parameters, that is, without sampling variability, to highlight the general properties of the criteria.

**Figure 1.**
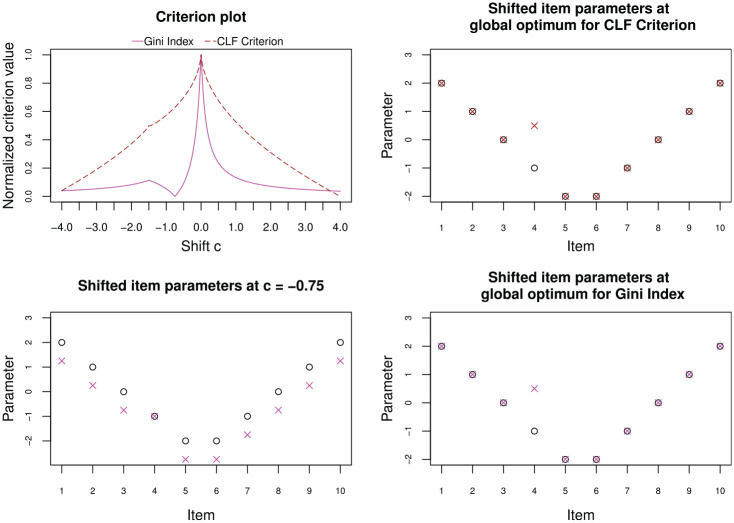
Criterion plot (top left), shifted item parameters according to global optima (right column) and shifted item parameters according to local maximum (bottom left) for toy example with one item displaying differential item functioning of size 0.75, based on true item parameters. *Note.* CLF = component loss function.

Figure 1 (top left) shows the criterion plot of the Gini Index and the CLF Criterion over a grid of values for possible shifts c.^4^ It shows that both the Gini Index and the CLF Criterion have their global optimum at the same shift value of 0. The item parameter locations that correspond to this global optimum are displayed in Figure 1 (right column, top for CLF Criterion, bottom for Gini Index). Both criteria agree on a solution where all items but the fourth item interlock, that is, only Item 4 shows DIF.

In the criterion plot in Figure 1 (top left), both criteria also show a smaller, local peak at the shift value −0.75, which is more notable for the Gini Index. The location of this second peak corresponds to a solution where the fourth item would interlock and all other items would show DIF, as illustrated in Figure 1 (bottom left). In this easy setting, both criteria—and the authors assume most readers—agree that the first solution, where Item 4 is labeled to have DIF while all other items have no DIF, is preferable. However, the authors will later show scenarios where the decision is not so clear cut.

Additional illustrations are provided in Online Appendix C.

## Simulation Studies

Now the results of two extensive simulation studies are presented, where the performance of the Gini Index and CLF Criterion are compared to each other and to that of the three anchoring methods from the literature that have been described above. For space constraints, here in the main text only a brief summary of the simulation setup and the key results are presented. All further details are provided in Online Appendix D.

### Simulation Study I

#### Simulation design

The simulation design for this first study was chosen to be very similar to that of Kopf et al. (2015a) to ensure comparability with this extensive comparison study. Data sets for two groups of subjects were simulated, the reference and the focal group, under the Rasch model. In most of the scenarios, a certain percentage of the items was simulated to show DIF between the groups. The direction of DIF was either balanced or unbalanced. There are also scenarios that were simulated completely under the null hypothesis with no DIF in any item. In each setting, 10,000 replications were simulated.

#### Results

In the following the authors will report the false alarm rate, that is computed as the percentage of items that were simulated as DIF free, but erroneously show a significant test result, and the hit rate, that is computed as the percentage of items that were in fact simulated to have DIF and correctly show a significant test result.

First the false alarm rates are checked in the null case scenario where no DIF items were generated. The false alarm rates should correspond to the nominal type I error rate of 5%. The results (omitted to save space) show that all methods roughly hold or fall below this nominal type I error rate in the null case scenario.

**Figure 2.**
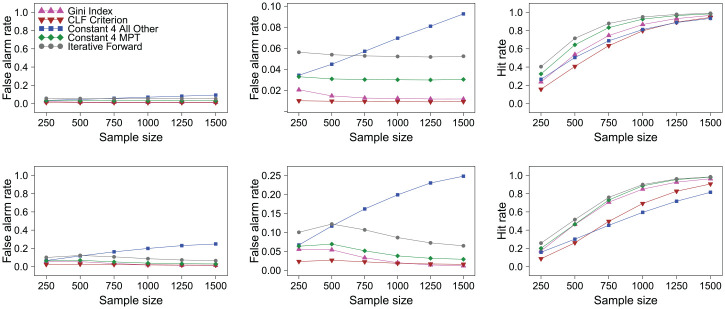
False alarm rates (y-axis from 0 to 1; left column), zoomed false alarm rates (y-axis from 0 to highest value; middle column) and hit rates (y-axis from 0 to 1; right column) for scenario with 20% (first row) and 40% (second row) differential item functioning items favoring the focal group. *Note.* CLF = component loss function; MPT = mean *p*-value threshold.

Now consider the results for scenarios with unbalanced DIF favoring one group. Figure 2 (first row) shows the false alarm rates, zoomed false alarm rates and hit rates for all methods in a scenario with a testlength of 40 items and 20% of these items being simulated with DIF. The results show that for this scenario all methods except for the “all other” method hold the nominal type I error rate. For the “all other” method, the false alarm rate notably increases with the sample size. This effect has already been discussed as a known problem of the “all other” method in unbalanced DIF settings in the introduction. All methods show hit rates that increase with the sample size as expected. The “iterative forward” method shows the highest hit rate, followed by the “constant four MPT” method, the Gini Index, the “all other” method, and the CLF Criterion.

When the percentage of DIF items rises to 40% in the next scenario displayed in Figure 2 (second row), it can be noted that the false alarm rates of some methods increase. Most notably, for the “all other” method, the false alarm rate increases even more strongly with an increasing sample size and goes up as high as 25%. For the other anchoring methods, as well as to a lesser degree for the Gini Index, we see a pattern where the false alarm rates show a slight inversely u-shaped pattern, which was similarly observed and explained by Kopf et al. (2015b). For the “iterative forward” method this also leads to a false alarm rate notably above the nominal 5% level for small and medium sample sizes, so that one should also interpret the hit rate of this method with caution. The hit rates of all methods increase with increasing sample size as expected. Again the “iterative forward” method shows the highest hit rate (but also an increased false alarm rate), followed by the “constant four MPT” method and the Gini Index, and with some distance by the CLF Criterion and the “all other” method.

In addition to the first two DIF scenarios, where a minority of 20% or 40% of the items were simulated with DIF, now a scenario is considered where the majority of items, 60%, are simulated with DIF in favor of the focal group. Since these items are simulated with DIF of the same amount, they work together as a cluster that is in itself invariant.

When one would stick to the definition of the simulation design for this setting, the false alarm rates for most methods would strongly increase, while the hit rates would decrease similarly dramatically, because the methods would consider the majority cluster as the DIF free one. However, as discussed above and further illustrated in Online Appendix D, from a philosophical point of view both solutions—considering the smaller or the larger item cluster as DIF free—are equally valid. The authors show in Online Appendix D that the Gini Index is particularly suited for identifying both solutions. Here they see a strong parallel to the works of Bechger and Maris (2015) and Pohl et al. (2017), who also critically discuss the general assumption that the majority of items is DIF free and instead aim at the detection of invariant item clusters.

**Figure 3.**
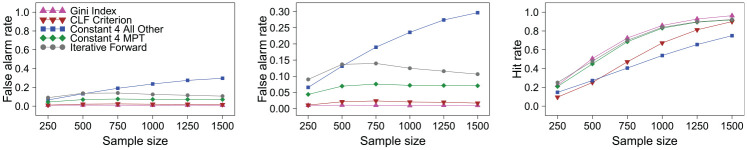
False alarm rates (y-axis from 0 to 1; left), zoomed false alarm rates (y-axis from 0 to highest value; middle), and hit rates (y-axis from 0 to 1; right) for scenario with 60 percent differential item functioning items favoring the focal group and label switching allowed. *Note.* CLF = component loss function; MPT = mean p-value threshold.

In the simulation study, where we need to decide on a scoring rule to be able to compute the aggregated false alarm rates and hit rates, this reasoning cannot be entirely transported, but one can try to mimic it by using a scoring rule that counts either solution as correct. The authors refer to this scoring rule as “label-switching,” because it resembles the fact that in cluster analysis one wants to judge whether observations correctly end up in the same cluster in two runs, but the labeling of the clusters is arbitrary. When this label-switching scoring rule is used for computing the false alarm rates and hit rates for all methods (Figure 3), the results return to what was seen for lower percentages of DIF items, namely that the methods show slightly increased (for the “iterative forward” method) or acceptable false alarm rates and increasing hit rates (except for the “all other” method, that has trouble with the unbalanced setting in general). The Gini Index now shows the highest hit rates, in particular notably higher than the CLF Criterion, as is further explained in Online Appendix D.

Online Appendix D also shows additional interesting results for the case of balanced DIF. In this setting, both Gini Index and CLF Criterion were outperformed by the traditional anchor selection methods, some of which are particularly well suited for balanced DIF. However, the findings for this setting also futher support the notion that the globally optimal solution does not tell the whole story, and that solutions corresponding to local optima in the criterion plot should also be explored to better understand the DIF structure in the data.

### Simulation Study II

#### Simulation design

To further illustrate the connection between DIF and multidimensionality, the authors have conducted a second simulation study, which employs a multidimensional IRT model for data generation. The design of this study resembles the design for unbalanced DIF in Simulation Study I as presented above. While there unidirectional DIF was generated by adding a fixed amount of DIF to certain item parameters, now the DIF is induced by letting certain items measure a secondary dimension in addition to the primary dimension (like described, e.g., in Roussos & Stout, 1996). For details, see again Online Appendix D. In each setting, again 10,000 replications were simulated.

#### Results

As expected, the results (displayed in Online Appendix D) were found to be very similar to those in Figures 2 and 3 for Simulation Study I, with only slightly higher false alarm and hit rates in some places. In particular, the methods are again able to identify the pattern in the items even when the majority of items measures the secondary dimension when the label-switching scoring rule is applied. The Gini Index, together with the “iterative forward” and “constant four MPT” methods, again performs particularly well in this setting.

## Empirical Application Examples

Online Appendix E provides two empirical application examples: one with a clear global optimum and one with an additional local optimum indicative of a DIF-inducing secondary dimension.

## Summary and Discussion

In this article, a new approach has been suggested for placing the item parameter estimates of a Rasch model for two groups of test takers on the same scale. The Gini Index, an inequality criterion from economics, has been suggested to be used as the optimization criterion, and its properties have been compared to those of the CLF Criterion, which is used in the alignment method by Asparouhov and Muthén.

It has been shown by means of extensive simulations, illustrative toy examples, and two application examples that the anchor point selection approach is able to identify locations on the item parameter continuum where the item parameter estimates for the two groups best overlap, and that there can be more than one sensible solution. Therefore, it is recommended that, rather than reporting only the globally optimal solution, the entire criterion plot should be reported and inspected, because it provides valuable additional information about the item structure. This information should be taken into account for the decision how to proceed with specific test items.

Asparouhov and Muthén (2014) have stated that

[. . .] [I]f data are generated where a minority of the factor indicators have invariant measurement parameters and the majority of the indicators have the same amount of noninvariance, the alignment method will choose the noninvariant indicators as the invariant ones, singling out the other indicators as noninvariant.

This corresponds exactly to the label-switching situations the authors have discussed above. However, they believe that, rather than considering this as a weakness of either approach, one should consider it as an advantage and utilize the information on multiple solutions contained in the criterion plots.

The recent article of Pokropek et al. (2020) investigates the effect of using different powers in the CLF—where Asparouhov and Muthén use a power of $\frac{1}{2}$ for the square root—and observe that for powers smaller than one, that show superior results in their study, local optima can occur. In their illustrations and simulation studies the authors have found local optima for both the Gini Index and the CLF Criterion, where those for the Gini Index were more distinct. While in many cases both criteria agreed on the global optimum, they also observed situations where this was not the case.

Depending on the simulation setting, the anchor point selection based on the Gini Index, which was suggested in this manuscript, performed equally well or even slightly better than existing anchor selection methods. However, both the Gini Index and the CLF Criterion were outperformed in the balanced DIF setting, for which some of the competitor methods are particularly well suited. Here it might also come into play that the mathematical results show that both the Gini Index and the CLF Criterion select single-item anchors in the framework considered here. Single-item anchors are more heavily affected by sampling error and the literature on anchor length implies that too short anchors diminish the power of the resulting DIF tests.

On the other hand, the fact that mathematically the set of possible solutions is limited to single-item solutions makes it computationally easily feasible to extend this approach, for example, to pairwise comparisons of multiple groups of test takers (such as several different language groups). In future research, the authors will also explore extensions to more general IRT models with different types of item parameters. Both extensions are possible for the Gini Index in the same way that is employed for the CLF criterion in Asparouhov and Muthén (2014) and Muthén and Asparouhov (2014).

It should also be noted that the illustrations of the results show that, despite mathematically corresponding to single-item anchors, all solutions represented as global or local optima in the criterion plot are well interpretable graphically and can help identify clusters of items representing, for example, DIF-inducing secondary dimensions.

An interesting line of future research would be to compare the results of the anchor point selection approach to approaches that explicitly aim at identifying item clusters, such as Bartolucci (2007), Pohl et al. (2017), Pohl and Schulze (2020), and Schulze and Pohl (2020). As already mentioned above, the approach of Pohl and colleagues, which is based on the work of Bechger and Maris (2015) and the notion of differences in relative item difficulties, is closely related in philosophy to the approach presented here. The authors would expect that the item clusters of Pohl and colleagues should largely agree with solutions corresponding to global or local optima, and believe that the Gini Index as an intuitive criterion, together with the possibility to graphically display the criterion plot, will be particularly helpful for test developers in understanding the patterns in their data and guiding their decision-making.

## Computational Details

The results were obtained using the R system for statistical computing (R Development Core Team, 2019), version 3.6.2. Anchor point selection will be made available in the R package psychotools. For the Gini Index, the authors used the implementation from the R package ineq (Zeileis, 2014), and for model fitting and DIF tests, they employed existing functionality from the R package psychotools (Zeileis et al., 2020). Simulation Study II used the R package mirt (Chalmers, 2012) for data generation.

## Supplemental Material

sj-pdf-1-apm-10.1177_0146621621990743 – Supplemental material for Anchor Point Selection Click here for additional data file. Supplemental material, sj-pdf-1-apm-10.1177_0146621621990743 for Anchor Point Selection by Carolin Strobl, Julia Kopf, Lucas Kohler, Timo von Oertzen and Achim Zeileis in Applied Psychological Measurement

## Notes

1. The literature on the anchor length shows that a too short anchor decreases the power of the following DIF tests, while a too long anchor increases the risk of a contaminated anchor (i.e., an anchor that includes DIF items), which can lead to artificial DIF (see also Andrich & Hagquist, 2012). An anchor length of three to five, most often four, items has been suggested as a compromise (cf. Egberink et al., 2015; Shih & Wang, 2009; Wang et al., 2012). Woods (2009) provides a more thorough discussion of anchor length choice, but this is not the focus of our study.

2. Note, however, that other estimation approaches, such as marginal maximum likelihood estimation, may make it necessary to account for possible impact through the specification of the person parameter distribution, which may also affect their sensitivity for detecting certain DIF patterns (cf., e.g., Debelak & Strobl, 2019).

3. Note that by definition, the Gini Index would be undefined in a case where all distances are exactly zero, because mathematically this would lead to a division by zero. In their implementation, the authors have redefined its value to zero in this case, because it also represents perfect equality.

4. The values on the y-axis have been normalized for both criteria to be able to compare their shape in one plot. Note also that in this and the following illustrations of the criterion plots, the authors use an extensive grid over the search interval for better visibility. In the simulation studies, on the other hand, they use the sparse grid described in Online Appendix F for computational efficiency, as it has been shown mathematically to contain all possible locations for optima.
