# Supplementary material for: Anchor Point Selection: Scale Alignment Based on an Inequality Criterion
Source: Appl Psychol Meas. 2021 Feb 25;45(3):214–30. doi: 10.1177/0146621621990743 (PMC8041453; doi:10.1177/0146621621990743)
Supplement: sj-pdf-1-apm-10.1177_0146621621990743 – Supplemental material for Anchor Point Selection [file sj-pdf-1-apm-10.1177_0146621621990743.pdf]

Online Appendices

Anchor point selection – Scale alignment based on an inequality criterion

Carolin Strobl, Julia Kopf, Lucas Kohler, Timo von Oertzen & Achim Zeileis

Contents

A Illustration of the effect of restrictions **1**

B Comparison of the shape of the criteria **3**

C Additional illustrations of the properties of the criteria **4**

D Simulation studies **7**

E Empirical application examples **19**

F Mathematical derivation of possible locations of optima **22**

References **24**

A Illustration of the effect of restrictions

The initial item parameter estimates are obtained using an arbitrary restriction, typically setting the first item parameter or the sum of all item parameters to zero. However, as is illustrated in Figure 1, if these initial item parameter estimates were naively used for a comparison between the two groups, the choice of the restriction would indeed affect our conclusion. This example was set up such that the first three item parameters are the same for both groups while the fourth item parameter differs between the groups. This is obvious in the first column a) of Figure 1, where in the top row a direct comparison of the item parameters and in the bottom row the setup of a graphical test (Rasch, 1960; Wright & Stone, 1999) is displayed. In this first column a), the first item parameter is arbitrarily set to 0 in both groups. In the top row of plots, an item displays DIF if the item parameters of the two groups (symbolized by circles and crosses) do not interlock. In the graphical test in the bottom row of plots, an item displays DIF if it is not located on the diagonal. (To account for estimation error, significance tests and confidence ellipses have been suggested for the graphical test, but here for simplicity we only focus on the location of the item parameters and act as if their true values were known.) Considering the selection of anchor

items, we see that item 1 was a good choice here because it shows no DIF itself and can be safely used to compare the other items.

In the second column b) of Figure 1, however, a different restriction was used: Here the sum of all item parameters was set to zero in both groups. In anchor terms, this would mean that all items were included in the anchor. Due to the DIF in item 4, this anchor is contaminated. When the scales are shifted according to this anchor, the between-group distance in item 4 decreases, but at the cost of all other items' distances increasing artificially.

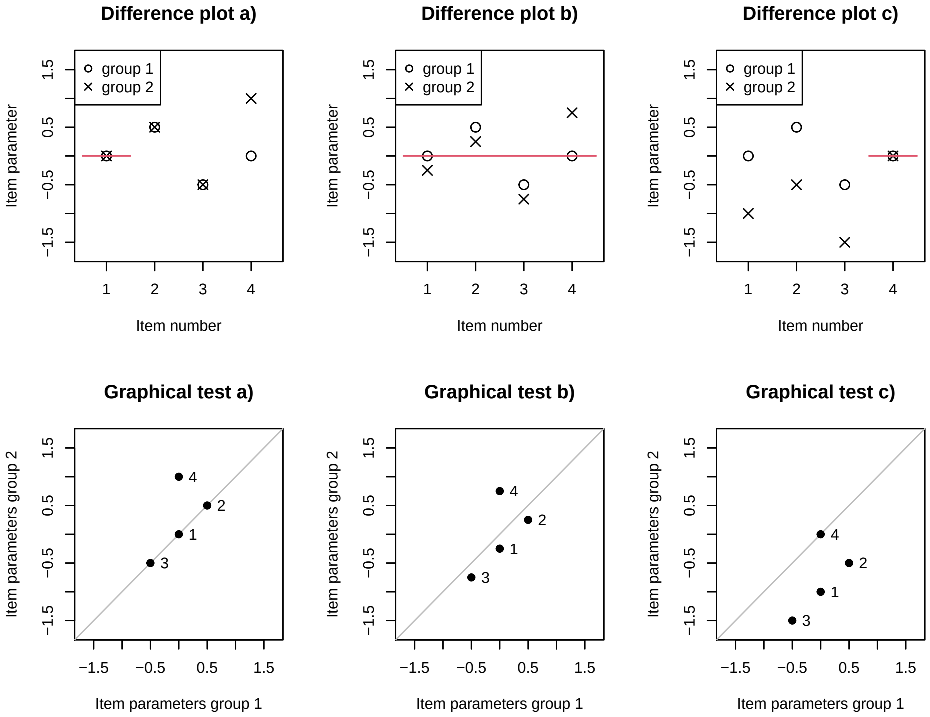

Figure 1: Illustration of comparisons of item parameters (top row) and graphical tests (bottom row) for different restrictions: a) $\beta_1^{(g)} = 0$, b) $\sum_j \beta_j^{(g)} = 0$, c) $\beta_4^{(g)} = 0$. Item numbers are displayed on the x-axis in the top row and next to the plotting symbols in the bottom row.

Even more extremely, when the parameter for item 4 is set to 0 in both groups in the third column c) of Figure 1, it looks like item 4 had no DIF, but all other items now exhibit the amount of DIF originally inherent in item 4. Most readers would agree that this is not a good choice and all traditional anchor selection approaches would try to avoid this scenario. However, had this been our initial arbitrary restriction for estimating the item parameters, and had we not investigated its effect, we could have come to a very different conclusion than before.

At this point it is important to note that our interpretation of which conclusion is right or wrong strongly depends on the abovementioned assumption that it is a minority of items that exhibit DIF, not the majority. Without any additional assumption, given the scale indeterminacy, it would not be possible to decide which scenario is the correct one.

As a side note, the didactically very well written textbook of Wright & Stone (1999) also implicitly follows this assumption. On p. 62 it shows an example of a graphical test where some items exhibit

DIF. There, the authors move the original identity line, that seems to have been based on the arbitrary restriction used for the item parameter estimation, towards the location of the majority of items. The "second identity line" of Wright & Stone (1999) is exactly what a sensible anchoring approach would produce in this situation (even if the authors do not yet use this terminology and it sounds like the line was manually placed through the "major item stream").

B Comparison of the shape of the criteria

Before the properties of both criteria for DIF detection are illustrated and compared to those of traditional anchoring approaches, let us display the shape of both criteria as a function of the between-group absolute distances $d_j(c)$ for two items in Figure 2. We can see that the two criteria have similar but not identical shapes: The Gini Index reaches its maximum (visible as "rooftops" in Figure 2, left) in all points where one item parameter has an absolute between-group distance of zero while the other item parameter has a value different from zero, which corresponds to the most inequal situation of one item being DIF free and the other item having "all the DIF" in a setup with only two items. On the diagonals, where the absolute between-group distances of the two items are equal, the Gini Index has the lowest values. Here the DIF would be equally distributed between all items, which would not be a reasonable choice for anchoring. This illustrates that the Gini Index behaves in a way that corresponds well to our intuition of DIF.

The CLF also shows relatively high values (visible as ridges of the "tent" in Figure 2, right) in points where one item parameter has an absolute between-group distance of zero while the other item parameter has a value different from zero, which corresponds to the most inequal situation of one item being DIF free and the other item having "all the DIF". Note, however, that the CLF Criterion produces higher values for solutions with lower absolute distances in the other item and does not decrease as strongly as the Gini Index on the diagonals where the absolute between-group distances of the two items are equal.

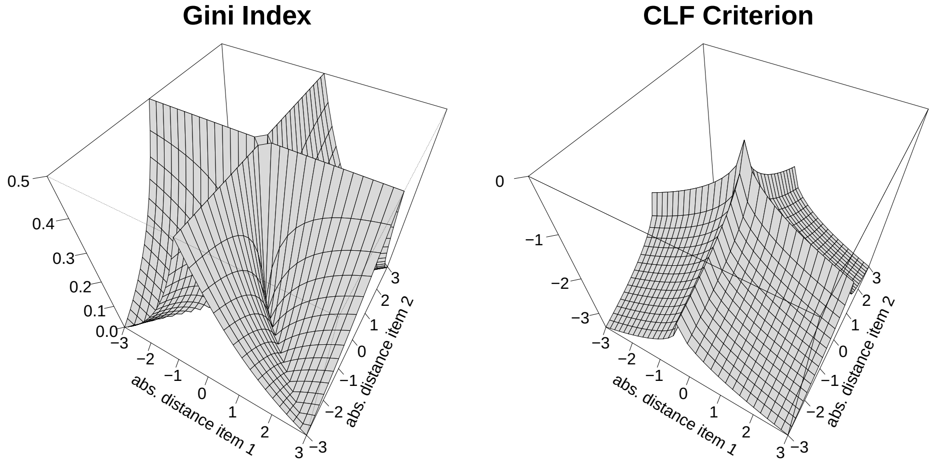

Figure 2: Shape of Gini Index and CLF Criterion for two items.

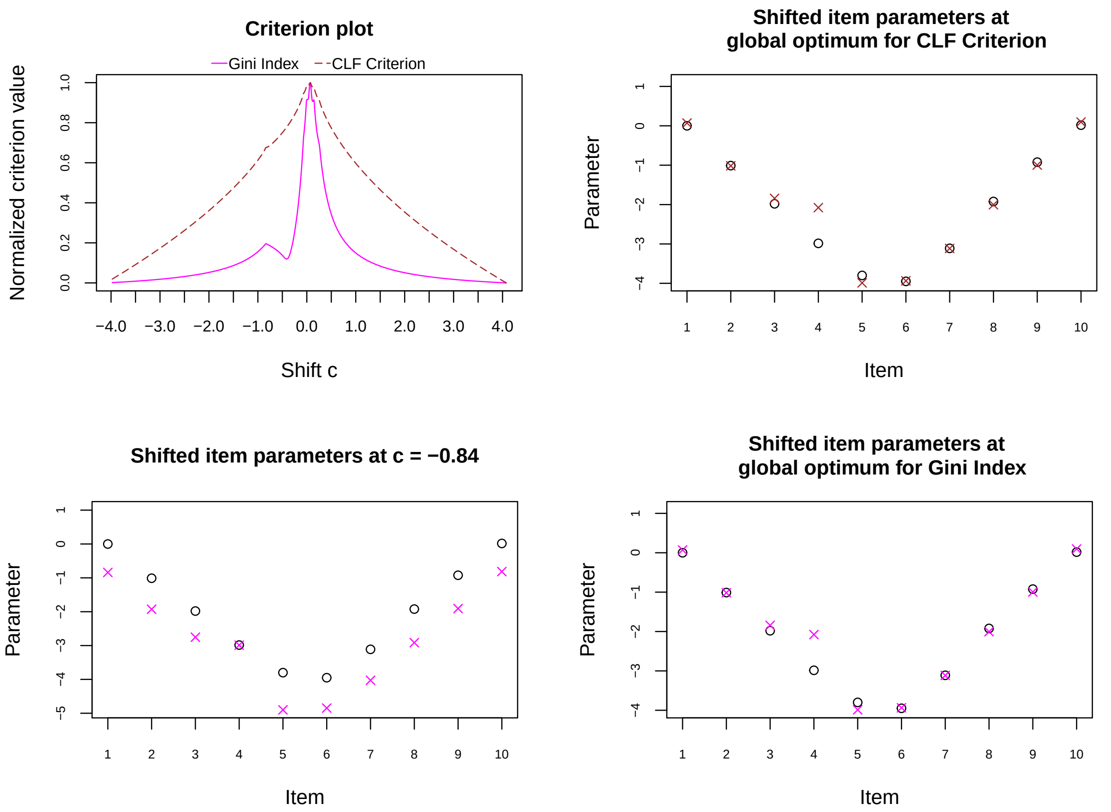

Figure 3: Criterion plot (top left), shifted item parameters according to global optima (right column) and shifted item parameters according to local maximum (bottom left) for toy example with one item displaying DIF of size 0.75, based on estimated item parameters.

C Additional illustrations of the properties of the criteria

In addition to the illustration with the true item parameters in the main text, we now simulate item response data from these item parameters and display the results again for the estimated parameters. This gives a more realistic impression of the effect of sampling variability. Figure 3 (top left) again shows the criterion plot of the Gini Index (pink) and the CLF Criterion (brown) over a grid of values for the shift c. Note that the plots are more shaky now in the region around the maxmim due to sampling variability, but otherwise show the same pattern as before. Also the locations of the item parameter estimates corresponding to the global optimum in Figure 3 (right column) are affected by sampling variability now and cannot all be perfectly aligned, but again they show a solution where all items but the fourth item roughly interlock. In addition, there is again a second peak, also slightly shifted in position due to sampling variability of the item parameter estimates, where the fourth item would interlock (Figure 3 bottom left).

With a similar toy example we would also like to illustrate the abovementioned property, that in principle the Gini Index is independend of the absolute amount of wealth – or in our case DIF: When we increase the amount of DIF in item 4 (1.5 vs. 0.75), we can see in Figure 4 that the first peak stays in the same position, but the second peak shifts to the position where the fourth item would interlock, $c = -1.5$, which is logical. What is not visible in this figure, however, is the raw values of the criteria at their

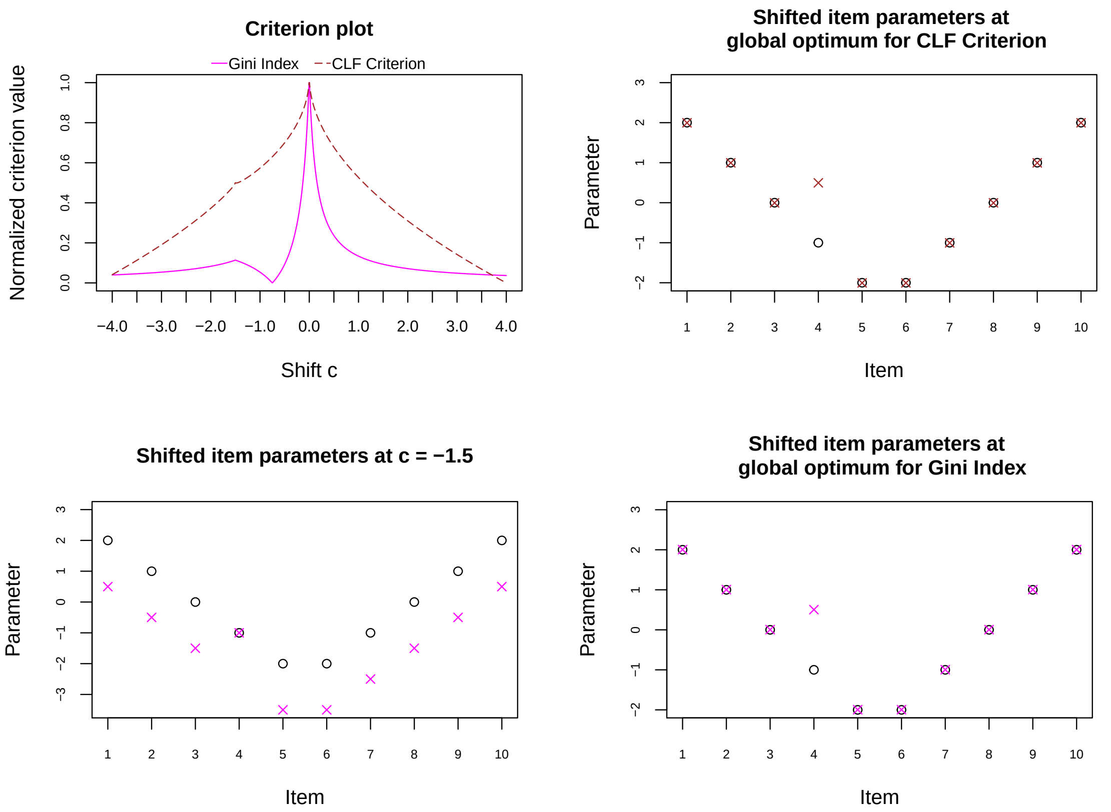

Figure 4: Criterion plot (top left), shifted item parameters according to global optima (right column) and shifted item parameters according to local maximum (bottom left) for toy example with one item displaying DIF of size 1.5, based on true item parameters.

optima, because they are normalized for comparability. We therefore display the raw values of the criteria for different DIF patterns in Table 1 and compare the first two rows of Table 1, where still only item 4 has DIF, and the amount of this DIF is doubled in the second row. We see that for the Gini Index it is only relevant that one out of ten items contains the entire amount of DIF. Its value does not vary with the amount of DIF that this item contains. This is different for the CLF Criterion, that does vary based on the amount of DIF. If, however, several items share the same overall amount of DIF, like in the third and fourth row of Table 1 (where the DIF effects add up to 1.5, but are distributed over two or three items respectively), both criteria show lower values when the same overall amount of DIF is distributed between more items.

DIF pattern	Gini Index	CLF Criterion
(0, 0, 0, 0.75, 0, 0, 0, 0, 0, 0)	0.90	−0.87
(0, 0, 0, 1.5, 0, 0, 0, 0, 0, 0)	0.90	−1.22
(0, 0, 0, 0.75, 0.75, 0, 0, 0, 0, 0)	0.80	−1.73
(0, 0, 0, 0.5, 0.5, 0.5, 0, 0, 0, 0)	0.70	−2.12

Table 1: Gini Index and CLF Criterion for different DIF patterns.

For completeness we also show what the criterion plots (Figure 5, left) and item parameter locations

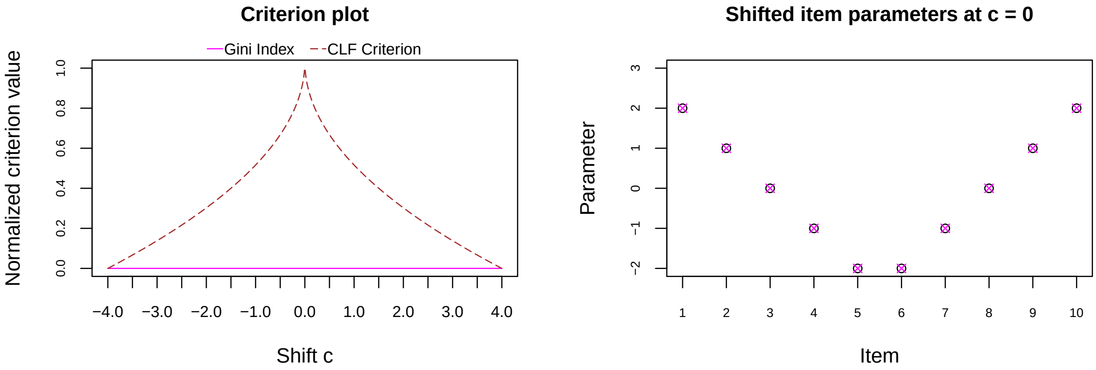

Figure 5: Criterion plot (left) and shifted item parameters according to global optimum (right) for toy example with no item displaying DIF, based on true item parameters.

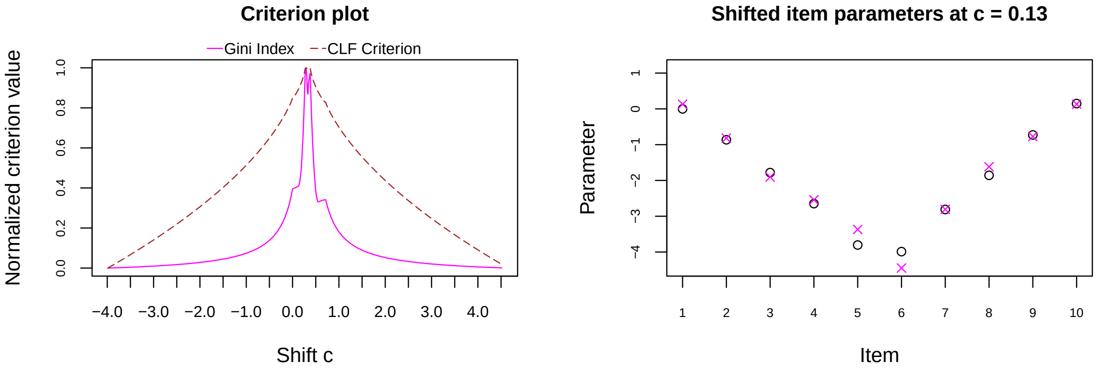

Figure 6: Criterion plot (left) and shifted item parameters according to global optima (right) for toy example with no item displaying DIF, based on estimated item parameters.

(Figure 5, right) look like when no DIF is present. In this setting, all items have a difference of zero between the groups. In this case, the Gini Index gives a flat criterion plot[1] and the CLF Criterion gives a single peak at the position where all differences between the item parameters are zero. Note, however, that this exact result is only possible when the true item parameters are used.

When we consider a more realistic situation and simulate item responses from the item parameters with no DIF, the criterion plot for the Gini Index looks quite different at first glance, as displayed in Figure 6 (left). Now we find that the criterion plot for the Gini Index also shows a clear peak because – due to the sampling variability – the item parameter estimates are no longer exactly identical between the two groups at any position. These empricial differences are treated by both criteria like items that show a small amount of DIF, so that again the optimal solution for both criteria is one where most items interlock as well as possible between the groups, but some items show small differences in their item parameter estimates – in this case due to the sampling variablity.

[1]Remember that originally the Gini Index would be undefined at the position in the center of the criterion plot, where all differences are exactly zero. We have re-defined it to be zero at this position, because it also represents a perfectly equal distribution. The plot for the item parameter locations (Figure 5, right) corresponds to this position in the center of the criterion plot. Since the resulting item parameter locations are exactly the same for Gini Index and CLF Criterion, they are only displayed once here to save space.

D Simulation studies

These simulation studies compares the performance of the Gini Index and CLF Criterion to each other and to that of three anchoring methods from the literature.

D.1 Simulation study I

The simulation design for this study was chosen to be very similar to that of Kopf, Zeileis & Strobl (2015a) to ensure comparability with this extensive comparison study.

D.1.1 Simulation design

We simulated data sets for two groups of subjects, the reference and the focal group, under the Rasch model. In most of the scenarios, a certain percentage of the items was simulated to show DIF between the groups, but there are also scenarios that were simulated completely under the null hypothesis with no DIF in any item. In each setting, 10000 replications were simulated.

Person and item parameters The person parameters were generated from a normal distribution with variance 1 and a mean of 0 for the reference group and of -1 for the focal group.

A set of 40 item parameters, that had been previously used by Wang, Shih & Sun (2012) and Kopf et al. (2015a), were the basis for our study design: β =(−2.522, −1.902, −1.351, −1.092, −0.234, −0.317, 0.037, 0.268, −0.571, 0.317, 0.295, 0.778, 1.514, 1.744, 1.951, −1.152, −0.526, 1.104, 0.961, 1.314, −2.198, −1.621, −0.761, −1.179, −0.610, −0.291, 0.067, 0.706, −2.713, 0.213, 0.116, 0.273, 0.840, 0.745, 1.485, −1.208, 0.189, 0.345, 0.962, 1.592). These item parameters were used for all settings with a test length of 40 items.

In order to be able to manipulate the test length, for settings with test lengths of 20 or 60 items, the respective number of parameters was randomly drawn from the original set of 40 values (in the case of 20 in random order without replacement, in the case of 40 in random order, and in the case of 60 in random order with replacement).

DIF-items Depending on the percentage of DIF items, the first test length $\times$ DIF percentage of the items were simulated to display DIF by means of setting the difference in the item parameters between reference and focal group to $+0.6$ or -0.6 depending on the intended direction of DIF.[2]

IRT model The item responses in each group were generated by means of the Rasch model.

Manipulated variables Similar to previous simulation studies such as Woods (2009); Wang et al. (2012) and Kopf et al. (2015a), the manipulated variables were the sample size, the test length, the direction of DIF, the percentage of DIF items and the anchoring methods.

Sample size The sample size for the simulated data sets was varied between 250 and 1500 in steps of 250. This overall sample size was divided equally between the two groups. (We also investigated settings with unequal samples sizes. For unequal group sizes the power was slightly diminished for all methods, but the comparisons between the methods were not affected by this factor. Therefore, in the interest of

[2]This is equivalent to a random assignment, because the item parameters are drawn in a random order, but simplifies the interpretation of the following figures.

saving space, we present only results for equal group sizes, which also makes the following plots easier to read.)

Direction of DIF and percentage of DIF items The direction of DIF is either balanced (where each DIF-item favors either the reference or the focal group, but no systematic advantage for one group remains because the effects cancel out), or unbalanced with an advantage for the focal group. (We have also investigated settings with an advantage for the reference group, but the results are virtually the same and thus not displayed in the interest of saving space.)

The percentage of DIF items relative to the overall test length was set to either 0%, 20%, 40% or 60%.

Anchoring methods The following methods were compared in this study:

- the "constant four all other" method suggested by Woods (2009),
- the "constant four MPT" method suggested by Kopf et al. (2015a),
- the "iterative forward" method suggested by Kopf, Zeileis & Strobl (2015b),
- anchor point selection based on the CLF Criterion employed by Asparouhov & Muthén (2014) and Muthén & Asparouhov (2014), and
- anchor point selection based on the Gini Index suggested in this paper.

Outcome variables Like in Kopf et al. (2015b) and Kopf et al. (2015a) the item-wise Wald test based on the conditional maximum likelihood item parameter estimates (cp. Glas & Verhelst, 1995; Kopf et al., 2015b) was used for the final DIF tests in our simulation studies. Note, however, as will become clear below, that our results are of a general nature that straightforwardly generalizes to other DIF tests.

Due to the fact that one restriction is necessary for the item parameter estimation, for a test length of m items only $m-1$ parameters can be estimated and tested freely. Therefore, one item cannot be formally tested for DIF in the final test. For the traditional anchor item selection methods, the item that was first selected into the anchor (and is thus considered the least likely to have DIF by the respective method) is not tested for DIF. For the anchor point selection approach, the item that shows the smallest item parameter difference between the groups in the global optimum of each criterion (again the one considered the least likely to have DIF by each criterion) is not tested for DIF.[3]

In the following plots we will report the average false alarm rate (that corresponds to the type I error) and the average hit rate (that corresponds to the power of the DIF tests) for the final DIF tests. The false alarm rate is computed as the percentage of items that were simulated as DIF free, but erroneously show a significant test result. The hit rate is computed as the percentage of items that were in fact simulated to have DIF and correctly show a significant test result.

[3]Note again that we show mathematically in Appendix F that in all local and global optima the item with the smallest item parameter difference actually perfectly interlocks and thus constitutes an anchor item. If DIF tests were to be carried out for other shift positions, e.g., for illustration purposes, our implementation would also treat the item with the least item parameter difference as the one that should not be tested.

D.1.2 Results

In the following, we will present the results for a test length of 40 items in all detail. The results for test lengths of 20 and 60 items showed very similar patterns and are thus omitted to save space. (For those results where the test length did have a small but notable effect, namely for the "constant four MPT" method and the "all other", this is mentioned below.)

Null case: No DIF First we have checked the false alarm rates in the null case scenario where no DIF items were generated. The false alarm rates should correspond to the nominal type I error rate of 5%. Our results (not displayed to save space) show that all methods hold this nominal type I error rate in the null case scenario, only the "iterative forward" methods shows a false alarm rate slightly above 5% in the scenario with a shorter test length of 20 items. The remaining methods show conservative false alarm rates (around 3%), with the CLF Criterion displaying the lowest (below 1%) and the Gini Index displaying the second lowest (around 2%) false alarm rate.

Unbalanced DIF Now we will look at the results for scenarios with unbalanced DIF favoring one group. Figure 7 (top row) shows the false alarm rates, zoomed false alarm rates and hit rates for all methods in a scenario with a testlength of 40 items and 20% of these items being simulated with DIF. The results show that for this scenario all methods except for the "all other" method hold the nominal type I error rate. For the "all other" method, the false alarm rate notably increases with the sample size. This effect is slightly more pronounced for shorter test lengths (results not shown for brevity) and has already been discussed as a known problem of the "all other" method in unbalanced DIF settings in the introduction. All methods show hit rates that increase with the sample size as expected. The "iterative forward" method shows the highest hitrate, followed by the "constant four MPT" method, the Gini Index, the "all other" method and the CLF Criterion.

When the percentage of DIF items rises to 40% in the next scenario displayed in Figure 7 (middle row), we note that the false alarm rates of some methods increase. Most notably, for the "all other" method, the false alarm rate increases even more strongly with an increasing sample size and goes up as high as 25%. For the other anchoring methods, as well as to a lesser degree for the Gini Index, we see a pattern where the false alarm rates show a slight inversely u-shaped pattern, that is more pronounced for longer test lengths (results not shown for brevity) and was similarly observed and explained by Kopf et al. (2015b). For the "iterative forward" method this also leads to a false alarm rate notably above the nominal 5% level for small and medium sample sizes, so that we should also interpret the hit rate of this method with caution. The hit rates of all methods increase with increasing sample size as expected. Again we find that the "iterative forward" method shows the highest hitrate (but also an increased false alarm rate), followed by the "constant four MPT" method and the Gini Index, and with some distance by the CLF Criterion and the "all other" method (that shows a lower increase in the hit rate for larger sample sizes, where it also showed a higher increase in the false alarm rate).

In addition to the first two DIF scenarios, where a minority of 20% or 40% of the items were simulated with DIF, we now consider a scenario where the majority of items, 60%, are simulated with DIF in favor of the focal group. The results in Figure 7 (bottom row) show that for this scenario, the false alarm

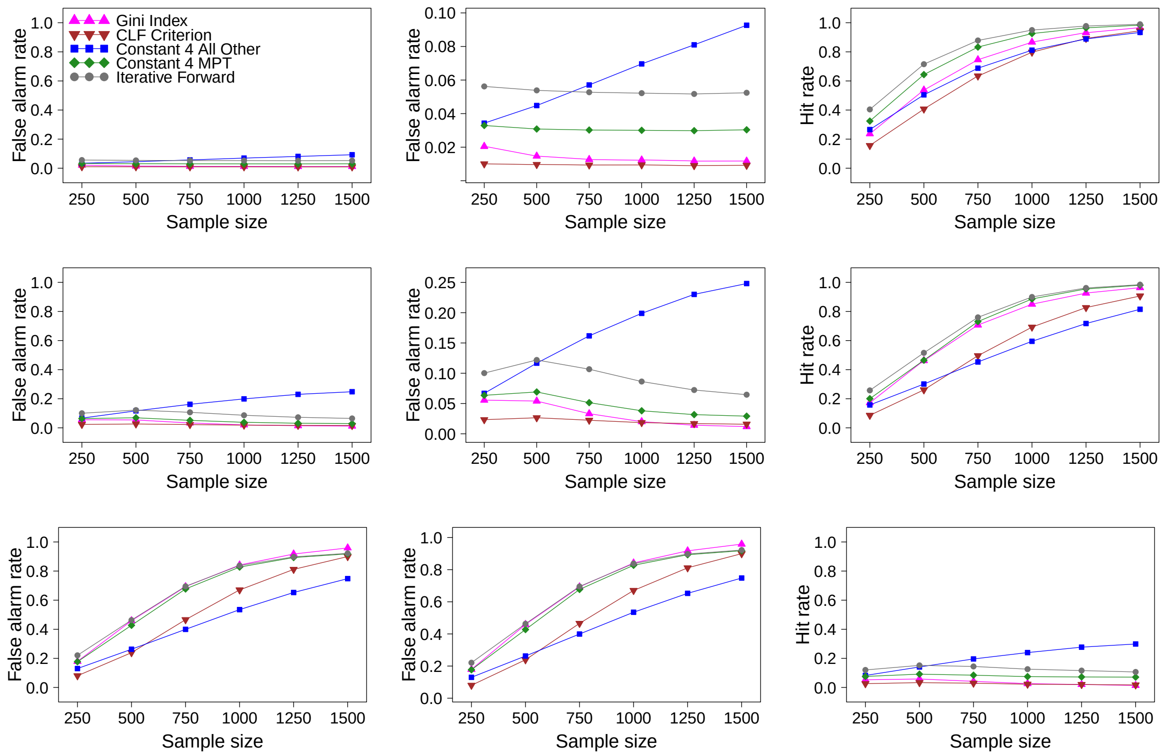

Figure 7: False alarm rates (y-axis from 0 to 1; left column), zoomed false alarm rates (y-axis from 0 to highest value; middle column) and hit rates (y-axis from 0 to 1; right column) for scenario with 20 percent (top row) 40 percent (middle row) and 60 percent (bottom row) DIF items favoring the focal group.

rates strongly increase, while the hit rates decrease similarly dramatically. (The only method that is less affected by this is the "all other" method, but we have seen above that this method also has its problems, they only work as an alleged advantage in this particular setting.)

To understand what is going on in this setting, Figure 8 (top left) illustrates the criterion plot for the item parameter values used in the simulations with 60% DIF. We find that the criterion plot has two peaks, corresponding to the displays of the shifted item parameters in Figure 8 (right column and bottom left).[4] At the global optimum (right column), the majority of items interlock. However, this majority of items has – by the definition of the simulation desing – been simulated with DIF of the same amount. This makes the items work together as the bigger cluster and results in the high false alarm rates in Figure 7 (bottom row). The second peak, on the other hand, where the smaller cluster of items interlocks, would be the correct one according to the simulation design. However, as we have discussed above, from a philosophical point of view both solutions can be equally valid. As we have discussed in the introduction, this is a situation where, since mathematically both solutions are equivalent, we have to decide based on considerations about the content of the items, and what the scale is supposed to measure, which solution is to be favored.

[4]Please note that in the simulations presented here, the item clusters always consist of neighboring items, because this makes them easier to detect visually. As will become clear from the empirical application examples, however, the methods work equally well when clusters are formed by non-neighboring items.

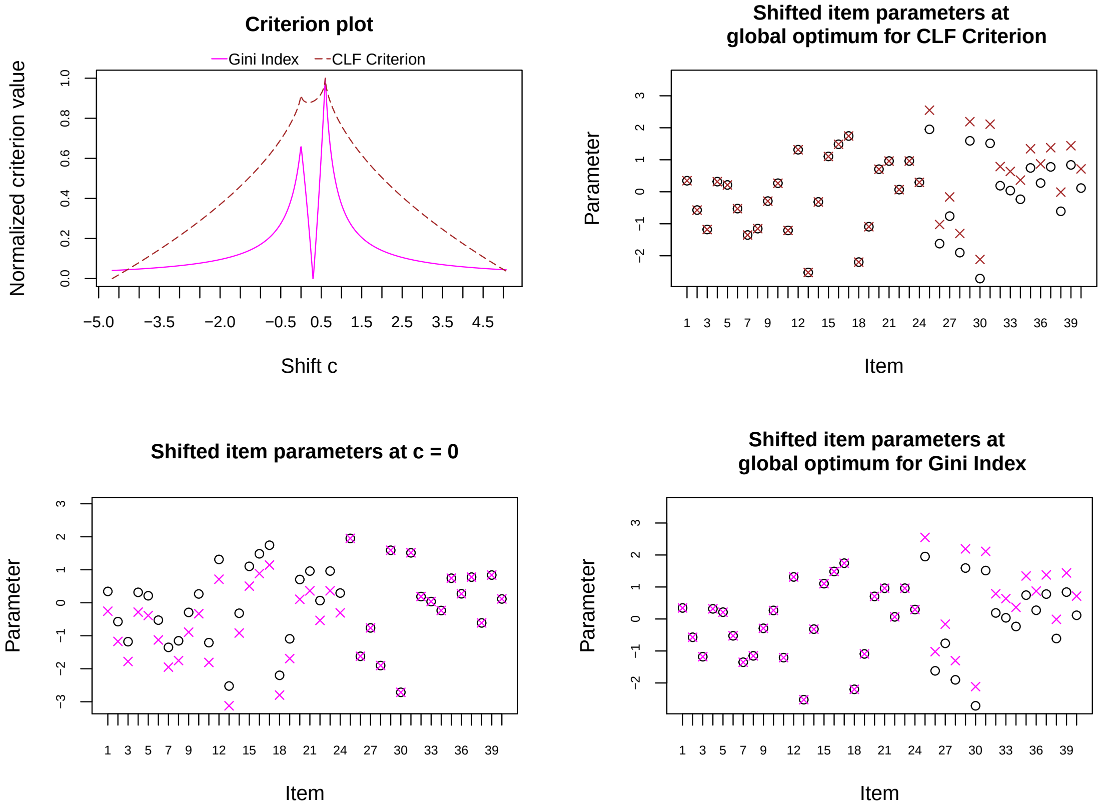

Figure 8: Criterion plot (top left), shifted item parameters according to global optima (right column) and shifted item parameters according to local maximum (bottom left) for simulation setting with 60 percent DIF items favoring the focal group, based on true item parameters.

Here we also see a strong parallel to the works of Bechger & Maris (2015) and Pohl, Stets & Carstensen (2017), who also critically discuss the general assumption that the majority of items is DIF free and aim at the detection of invariant item clusters. In our approach, multiple invariant item clusters correspond to multiple local optima in the criterion plot.

Let us first look again at the results where we stick to the definition of the simulation design and label the minority of items as DIF free and the majority of items as having DIF. The results for this view are displayed in Figure 7 (bottom row). We see that all anchoring methods show an increased false alarm rate now, because they tend to label the majority of items to be DIF free, which is counted as wrong under this view. Notably, the “all other” method, that showed an increased false alarm rates in the earlier settings, now has the lowest false alarm rate for larger sample sizes. The reason for this is that this method assumes that DIF was balanced, so that it selects a solution closer to the simulation design. Similarly, the CLF Criterion shows a lower false alarm rate than the other methods for reasons we will shortly illustrate.

Considering the hit rates (Figure 7, bottom row, right panel) we see that now the methods do not improve with increasing sample size (except for the “all other” method, that improves as an artifact of its erroneous assumption of balanced DIF). From the pattern we notice that the hit rates in this setting (with 60% DIF items) follow the same pattern as the false alarm rates in the earlier setting (with 40% DIF items), because the methods consider the majority of items as DIF free and accordingly mislabel

the other items.

Let us further explore why the false alarm rates of the CLF Criterion were apparently also less affected by this extreme scenario: When we look at the criterion plots for the Gini Index and the CLF Criterion (based on the simulated items parameters from Figure 8, top), we find that for the CLF Criterion the two peaks corresponding to the two solutions have more similar criterion values than for the Gini Index. Therefore, in the simulations from this setting with random variation, the CLF Criterion may be more likely to pick the left peak in some simulation runs (an exemplary illustration of one simulation run, where for the CLF Criterion the empirical global optimum corresponds to the left peak, is provided in Figure 9). Since picking the left peak corresponds to labeling the minority of the items as DIF free – which is against our intuition but in line with the scoring rule currently considered – the CLF Criterion is less affected in its false alarm rate and hit rate in Figure 7.

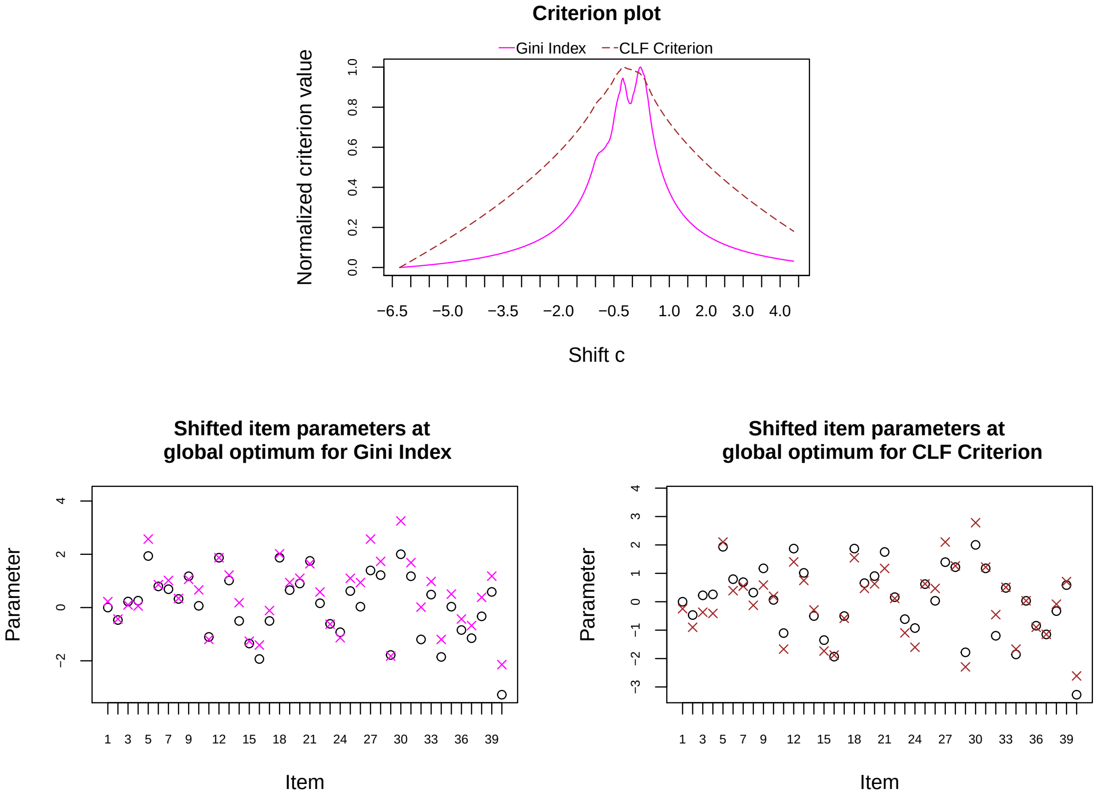

Figure 9: Criterion plot (top) and shifted item parameters according to global optima (bottom) for simulation setting with 60 percent DIF items favoring the focal group, based on estimated item parameters.

We argue throughout this manuscript that this additional information provided by the criterion plot is extremely valuable and should be taken into account – together with the item content and the question what the scale is supposed to measure – when deciding which items should be labeled as DIF items and how to proceed with this information. In the simulation study, where we need to decide on a scoring rule to be able to compute the aggregated false alarm rates and hit rates, this reasoning cannot be entirely transported, but we can try to mimic it by using a scoring rule that counts either solution as correct. This scoring rule counts both the solution where the cluster of items labeled as DIF items in the

simulation design is identified as DIF items, and the solution where exactly the other cluster of items is identified as DIF items as correct, but still counts any item assignment not corresponding to either of the two solutions as wrong. We refer to this scoring rule as “label-switching”, because it resembles the fact that in cluster analysis we want to judge whether observations correctly end up in the same cluster in two runs, but the labeling of the clusters is arbitrary.

When this label-switching scoring rule is used for computing the false alarm rates and hit rates for all methods (Figure 10), we see that the results return to what we saw for lower percentages of DIF items, namely that the methods show slightly increased (for the “iterative forward” method) or acceptable false alarm rates and increasing hit rates (except for the “all other” method, that has trouble with the unbalanced setting in general). The Gini Index now shows the highest hit rates, in particular notalby higher than the CLF Criterion. We assume that this is due to the fact that the criterion plot of the Gini Index more clearly distinguishes between the two peaks, which under the label-switching scoring rule both result in low false alarm rates and high hit rates, whereas the CLF Criterion shows similarly high criterion values for the entire peak area in Figure 9 (top), so that it may be more likely in a simulation with random variability to select a shift value in between, that corresponds to neither one of the two correct solutions.

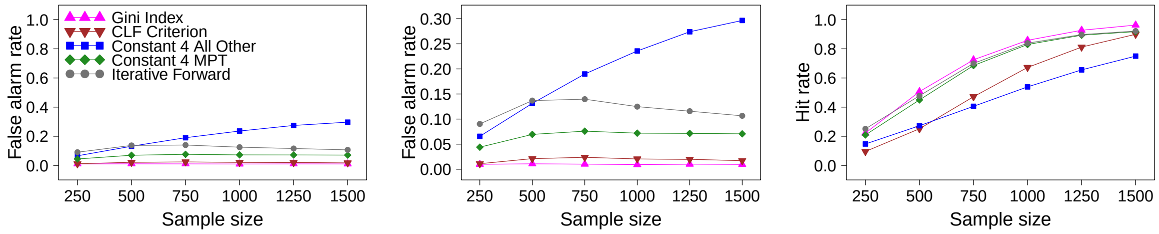

Figure 10: False alarm rates (y-axis from 0 to 1; left), zoomed false alarm rates (y-axis from 0 to highest value; middle) and hit rates (y-axis from 0 to 1; right) for scenario with 60 percent DIF items favoring the focal group and label switching allowed.

Balanced DIF Next we will look at the results for scenarios with balanced DIF, where half of the DIF items favor the reference and half of the DIF items favor the focal group.

Figure 11 (top row) shows the false alarm rates, zoomed false alarm rates and hit rates for all methods in a scenario with a testlength of 40 items and 20% of these items being simulated with balanced DIF. The results show that for this scenario all methods roughly hold the nominal type I error rate and show hit rates that increase with the sample size as expected. The “iterative forward” method again shows the highest hitrate (but note that it also shows a slightly increased false alarm rate), followed by the the “all other” method, the “constant four MPT” method, and, with some distance, the Gini Index and the CLF Criterion. A very similar picture can be found in Figure 11 (middle row) for 40% DIF items.

When the DIF percentage is increased to 60% in Figure 11 (bottom row), however, we notice that the Gini Index shows a strongly inflated false alarm rate and a diminished hit rate, while the other methods, including the CLF Criterion, are not as much affected. These results may at first look surprising, but can also be well explained when we again look at the simulated item parameters and the criterion plot

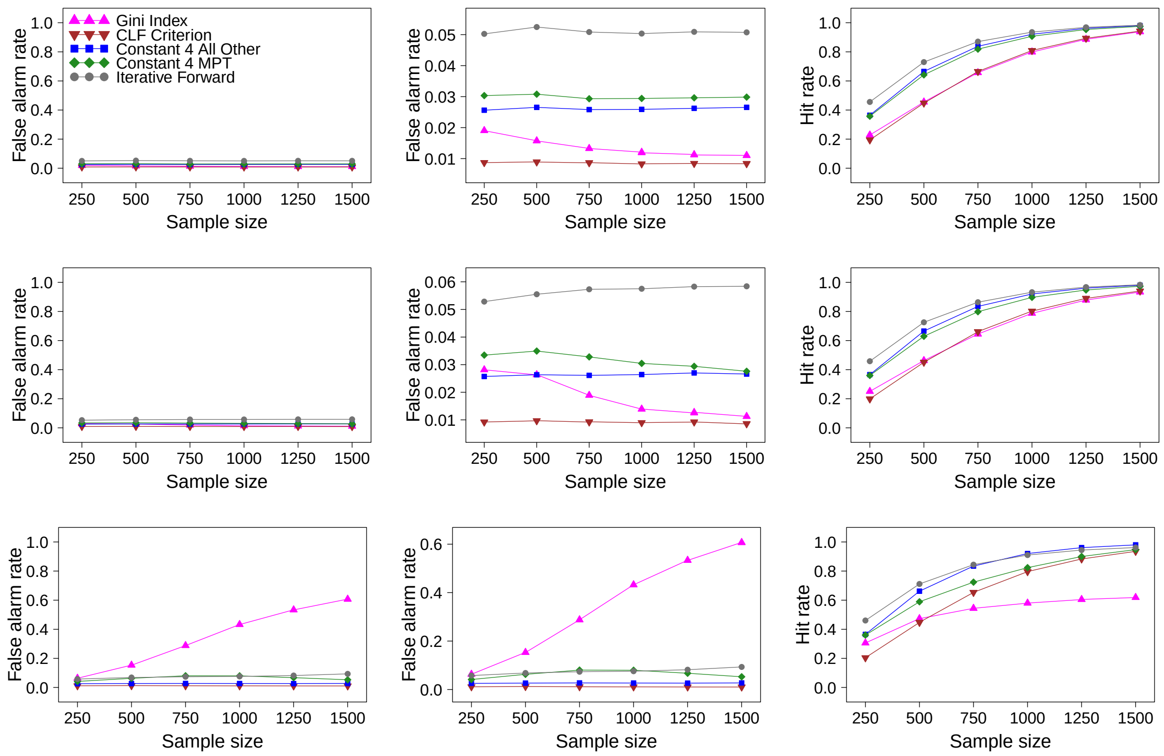

Figure 11: False alarm rates (y-axis from 0 to 1; left column), zoomed false alarm rates (y-axis from 0 to highest value; middle column) and hit rates (y-axis from 0 to 1; right column) for scenario with 20 percent (top row) 40 percent (middle row) and 60 percent (bottom row) DIF items in balanced setting.

in Figure 12.

What is important to understand this simulation scenario is that in the case of 60% DIF items in the balanced setting, the items actually form three clusters: 30% of the items are simulated with DIF favoring the reference group, 30% with DIF of the same size favoring the focal group, and 40% of the items are simulated without DIF. One solution for shifting the item parameters would recover this pattern, with the largest cluster of 40% of the items interlocking. This solution corresponds to the central peak in the criterion plot in Figure 12 (top). We see that the CLF Criterion has the highest peak at this solution. The item parameter locations corresponding to this solution are displayed in Figure 12 (bottom right), where we see that the the items in the largest cluster (on the right hand side of the plot) interlock. Due to the high central peak of the CLF criterion plot, this solution will be found in most simulation runs. Therefore, this solution leads to non-increased false alarm rates under the original scoring rule for the CLF Criterion.

The Gini criterion, on the other hand, also shows two clearly distinguishable peaks for the other two solutions, where either one of the clusters containing 30% of the items interlock, and a slightly lower central peak. Over the simulation runs with random variability, it will jump back and forth between all three solutions, in many cases selecting not the central peak but one of the other two solutions, one of which is displayed in Figure 12 (bottom left). However, in the original scoring rule underlying Figure 11 (bottom row), either of these solutions is counted as wrong. This causes the strongly increased false alarm rate of the Gini Index in this setting. It even increases with sample size because on average

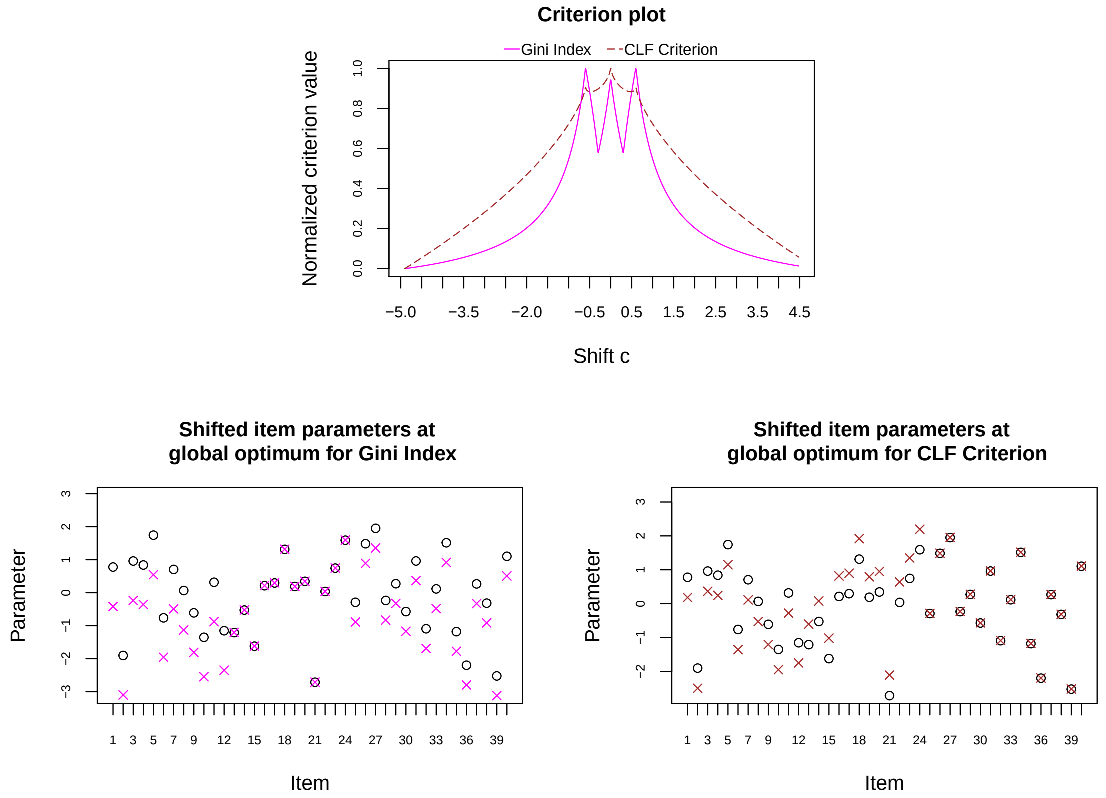

Figure 12: Criterion plot (top) and shifted item parameters according to global optima (bottom) for simulation setting with 60 percent DIF items in a balanced setting, based on true item parameters.

the peaks become more distinguishable as sample size increases and item parameter estimates are less variable.

If, however, we again adapt our view and count either of the three possible solutions as correct by means of using the label-switching scoring rule for all methods, we see in Figure 13 that the false alarm rate of the Gini Index returns to a very low value (while the "iterative forward" and "constant four MPT" methods show slightly increased false alarm rates), and the hit rate of the Gini Index is now close or equal to that of the CLF Criterion.

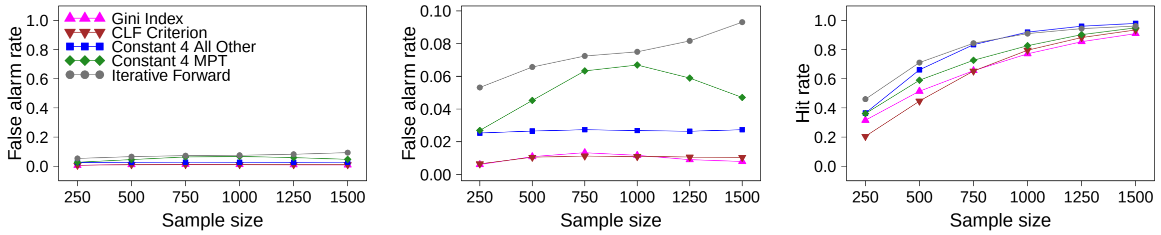

Figure 13: False alarm rates (y-axis from 0 to 1; left), zoomed false alarm rates (y-axis from 0 to highest value; middle) and hit rates (y-axis from 0 to 1; right) for scenario with 20 percent DIF items in balanced setting with label switching allowed.

To shed further light on the different behaviors of the CLF Criterion and Gini Index for this setting, we will increase the DIF percentage even further. This will highlight how both crireria treat different

combinations of item cluster sizes and item parameter differences.

When we produce an even more extreme setting with 70% of the items being simulated with balanced DIF, this results again in three item clusters, now with 35% of the items favoring the reference group, 30% neutral items, and 35% of the items favoring the focal group. In this scenario (Figure 14, left; only the criterion plot is presented to save space) the item cluster corresponding to the central solution is the smallest cluster, which makes the other two solutions even more plausible alternative solutions. The Gini Index, that already showed higher peaks for these two solution before, now shows this pattern even more pronounced. Interestingly, however, the CLF Criterion still produces the highest peak for the central solution, which labels the smallest item cluster as DIF free at the cost of labelling the two other item clusters as having DIF. This was an advantage in the simulation study with the original scoring rule, but does not well reflect our intuition.

Even if the three groups were of exactly equal sizes (results not shown for brevity) we would find a similar pattern for the criterion plot as in Figure 14 (left), with the CLF Criterion preferring the central peak and the Gini Index preferring the other two solutions.

Figure 14 (right) further illustrates this finding: On the x-axis it displays the percentage of items in the two non-central clusters taken together. On the y-axis it displays the relative height difference between the central peak and the non-central peaks for each criterion (as a percentage of the height difference for the minimum possible amount of balanced DIF for each criterion, to allow comparability). Values above zero (i.e., above the black horizontal line) indicate that the criterion prefers the central solution for a given percentage of items, while values below zero (i.e., below the black horizontal line) indicate that the criterion prefers the non-central solutions. While the Gini Index prefers the non-central solutions already at a percentage close to 60% (corresponding to cluster sizes of 30%, 40% and 30%), the CLF criterion still prefers the central solution when the percentage is close to 80% (corresponding to cluster sizes of 40%, 20% and 40%).

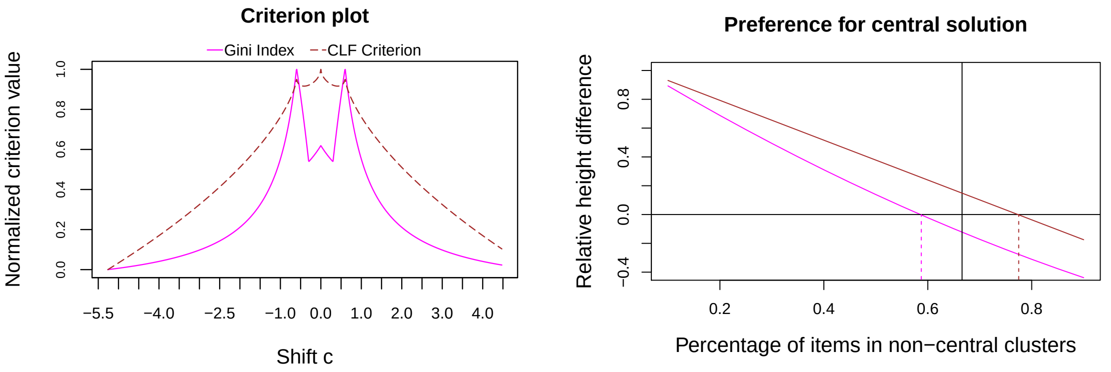

Figure 14: Criterion plot for simulation setting with 70 percent DIF items in a balanced setting, based on true item parameters (left) and illustration of preference for central solution as a function of the item cluster sizes (right).

Intuitively, one could argue that a neutral criterion without any additional assumptions should treat all three solutions as equal when the cluster sizes and DIF amounts are the same. This rationale is

represented in Figure 14 (right) by the vertical black line at 66% (corresponding to equal cluster sizes of 33% each). This illustration shows that neither of the two criteria investigated here corresponds exactly to this rationale, but that the behavior of the Gini Index is somewhat closer to it. Over a certain range of percentages, the Gini Index aready prefers the non-central solutions, where the DIF of the non-interlocking items goes in the same direction, while the CLF Criterion keeps preferring the central solution, where the DIF in the non-interlocking items cancels out. With respect to the optimal solutions it generates, this behavior of the CLF Crierion resembles the assumption of balanced DIF in traditional anchoring methods. For practical decisions derived from either crierion, these findings again support our notion that the globally optimal solution does not tell the whole story, and that solutions corresponding to local optima in the criterion plot should also be explored.

D.2 Simulation study II

In order to further illustrate the connection between DIF and multidimensionality, we have conducted a second simulation study, that employs a multidimensional IRT model for data generation.

D.2.1 Simulation design

The design of this study resembles the first part of Simulation Study I for unbalanced DIF. While there unidirectional DIF was generated by adding a fixed amount of DIF to certain item parameters, now the DIF is induced by letting certain items measure a secondary dimension in addition to the primary dimension (like described, e.g., in Roussos & Stout, 1996).

Person and item parameters The person parameters were generated from a bivariate normal distribution with means of 0.5 and 0.5 in the reference group and - 0.5 and -0.5 in the focal group, a variance of 1 for each dimension in each group and a covariance of 0.5 between the two dimensions in each group. A set of 40 intercept parameters was randomly drawn from a normal distribution with a mean of 0 and a standard deviation of 0.5. All items measured the first dimension with a fixed discrimination of 1.

DIF-items Depending on the percentage of DIF items, the first test length $\times$ DIF percentage of the items also measured the second dimension with a fixed discrimination of 1. Given that the person parameter distributions (in this simple case only the means) on the secondary dimension differed between the groups, this induces (uniform) DIF in these items (for further details see Roussos & Stout, 1996; Ackerman, 1992).

IRT model The item responses in each group were generated by means of a compensatory dichotomous multidimensional IRT model with the abovementioned specifications (for details on the generating model see Reckase, 2009; Chalmers, 2012), which corresponds to the multidimensional counterpart of a Rasch model.

Manipulated variables Similar to Simulation Study I and previous studies, the manipulated variables were the sample size and the percentage of DIF items, with the same levels as in Simulation Study I. In each setting, again 10000 replications were simulated.

D.2.2 Results

As expected, we find the results in Figures 15 and 16 to be very similar to those in Figures 7 and 10 for Simulation Study I, with slightly higher false alarm and hit rates in some places but the same general results, that have already been described in detail above.

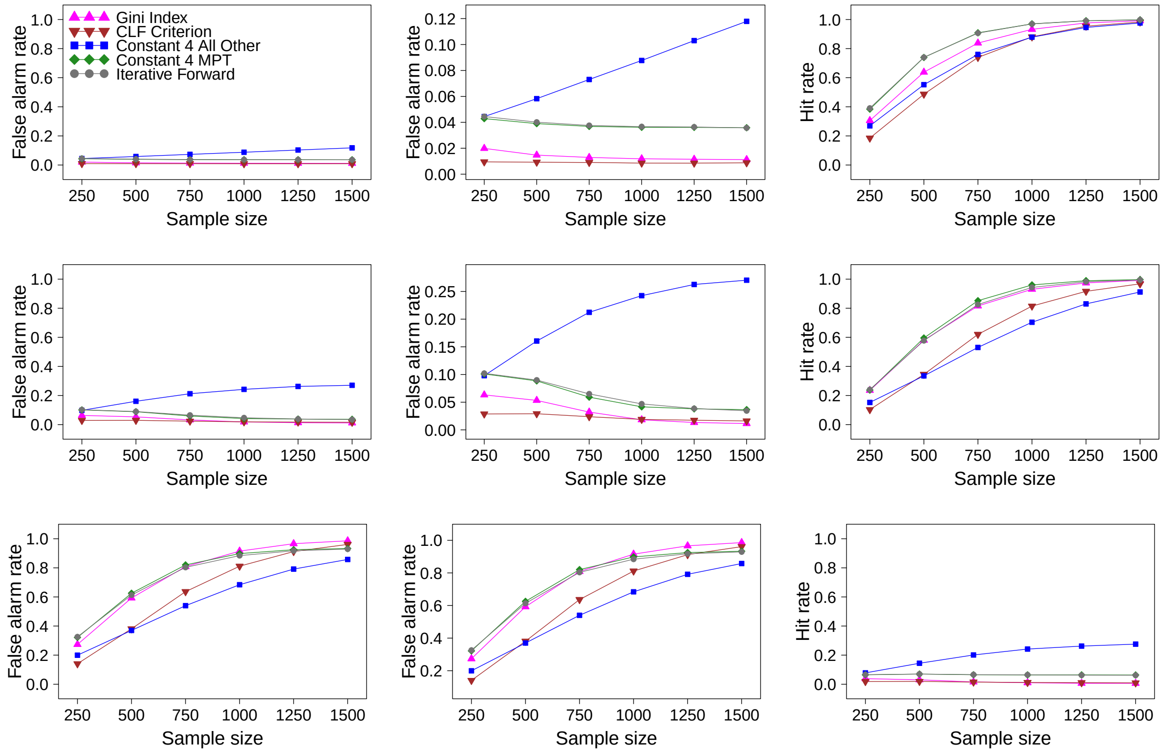

Figure 15: False alarm rates (y-axis from 0 to 1; left column), zoomed false alarm rates (y-axis from 0 to highest value; middle column) and hit rates (y-axis from 0 to 1; right column) for scenario with 20 percent (top row) 40 percent (middle row) and 60 percent (bottom row) DIF items induced by a secondary dimension.

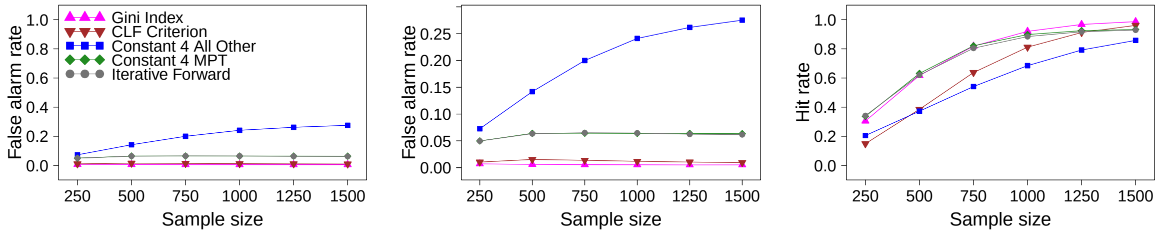

Figure 16: False alarm rates (y-axis from 0 to 1; left), zoomed false alarm rates (y-axis from 0 to highest value; middle) and hit rates (y-axis from 0 to 1; right) for scenario with 60 percent DIF induced by a secondary dimension and label switching allowed.

In particular, we find that when 60% of the items measure the secondary dimension, the original scoring rule again results in very low hit rates (Figure 15, bottom). With the label-switching scoring rule (Figure 16), however, we can see that the methods are again able to identify the pattern in the items

with increasing sample size, and that the Gini Index, together with the "iterative forward" and "constant four MPT" methods, again performs particularly well in this setting.
Plotting a criterion plot (not shown to save space) for this setting would also show two peaks here, similar to the one displayed in Figure 9, because again by design the items form two clusters.

E Empirical application examples

For further illustration the anchor point selection method will be applied to two empirical data sets from an online quiz for testing one's general knowledge and from a personality item pool.

E.1 Application example I: General knowledge quiz

An online quiz for testing one's general knowledge was conducted by the German weekly news magazine DER SPIEGEL in 2009. Overall, about 700,000 respondents participated in this general knowledge quiz and also answered a set of sociodemographic questions. The quiz consisted of a total of 45 items from five different domains: politics, history, economy, culture, and natural sciences. For each domain, four different sets of nine items were available, that were randomly assigned to the participants. A thorough discussion and analysis of the original data set is provided in Trepte & Verbeet (2010).
Here we consider an exemplary sample of university students enrolled in the federal state of Bavaria, who had been assigned questionnaire number 20. This sample contains 1075 cases (417 male and 658 female) and is freely available in the `psychotree` R package (Zeileis, Strobl, Wickelmaier, Komboz & Kopf, 2018), where also the wording of all 45 items contained in this quiz is documented.
The result of the anchor point selection for the general knowledge quiz data is depicted in Figure 17. From the criterion plot (Figure 17, left), we can see that there is a clear global maximum for both the Gini Index and the CLF Criterion, and only a slight second bump.[5] When we display the shifted item parameters at the global optimum (Figure 17, right), we see that some item parameters interlock visibly between the groups, while others show smaller or larger distances. To judge which of these distances correspond to significant DIF, the symbols for the item parameters in the male group are colored according to the results of the Wald test, with items marked in red displaying significant DIF and items marked in green not displaying significant DIF.
We did not account for multiple testing here, so that the results should not be overinterpreted. However, when we look at those items that exhibit the largest amount of DIF (indicated in Figure 17, right, by yellow highlighting) we find that item 19 ("Who is this? - Picture of Dieter Zetsche, CEO of Mercedes-Benz.") shows a higher difficulty for female participants, whereas items 40 ("What is also termed Trisomy 21? - Down syndrome.") and 43 ("Which kind of bird is this? - Blackbird.") show a lower difficulty for female participants. It is plausible that these items exhibit DIF with respect to the variable gender, for example because they are of differently high interest for male and female participants.

[5] The slight second bump is caused by a small group of items that in the globally optimal solution show a lower difficulty for female participants to a similar degree, and thus also form a small cluster.

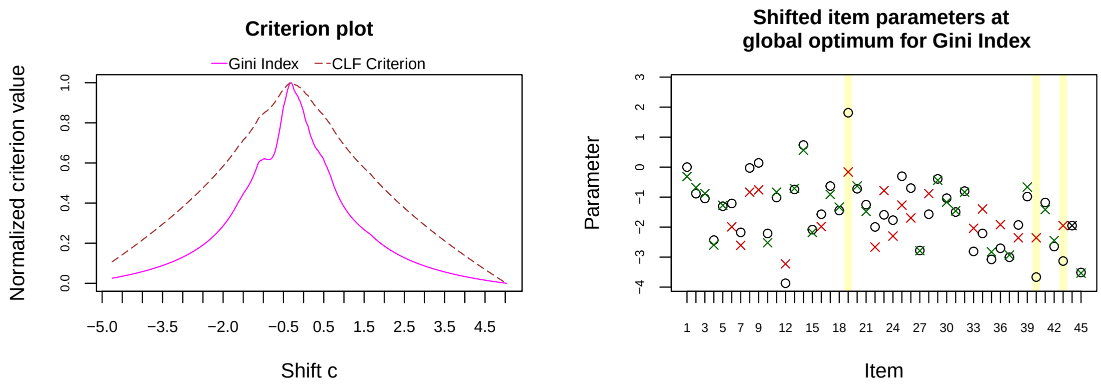

Figure 17: Criterion plot (left) and shifted item parameters according to global optimum (right), with items displaying statistically significant DIF indicated in red and items not displaying statistically significant DIF indicated in green, based on estimated item parameters for the general knowledge quiz data. The item parameters symbolized by circles belong to the female group, those symbolized by crosses to the male group. Items indicated by yellow highlighting are referred to in the text.

E.2 Application example II: International personality item pool

In this second example we would like to highlight again the notion of DIF induced by an unaccounted secondary dimension, on which the groups differ. For this example, we use data from 2800 subjects (1881 female and 919 male), taken as a subsample from the International Personality Item Pool (ipip.ori.org) data. This data set is freely available in the `psych` R package (Revelle, 2018), where also the wording of all items is documented.

For didactic reasons we include only the first three factors, Agreeableness, Conscientiousness and Extraversion. Each factor is measured by five items, some of which are inversely phrased.[6] The item responses were originally encoded using a six point scale ranging from *very inaccurate* to *very accurate*. For this exemplary analysis with the binary Rasch model, the responses have been recoded to a binary format, with the three lower categories being recoded as 0 and the three upper categories being recoded as 1.

From the criterion plot in Figure 18 (top), we can see that there is again one dominant global maximum for both the Gini Index and the CLF Criterion, but – in contrast to the first application example – now we also see a clearly pronounced second peak to the right of the global optimum, again more notably in the criterion plot for the Gini Index. The shifted item parameters at both these locations are shown in Figure 18 (bottom, left for global, right for local optimum). As in the previous section, items marked in green do not show significant DIF, while items marked in red do show significant DIF according to the Wald test.

Given that the first 15 self report items are supposed to cover three different factors, that we inappropriately placed on one joint scale, we might have expected to encounter three clusters of items based on these dimensions. That could have been the case if the two groups, males and females, would show

[6] For the remaining factor Neuroticism there are no inversely phrased items. For Opennness the two inversely phrased items do not function in the same way that we will see for the first three factors, possibly because their content does not induce gender specific social desirability.

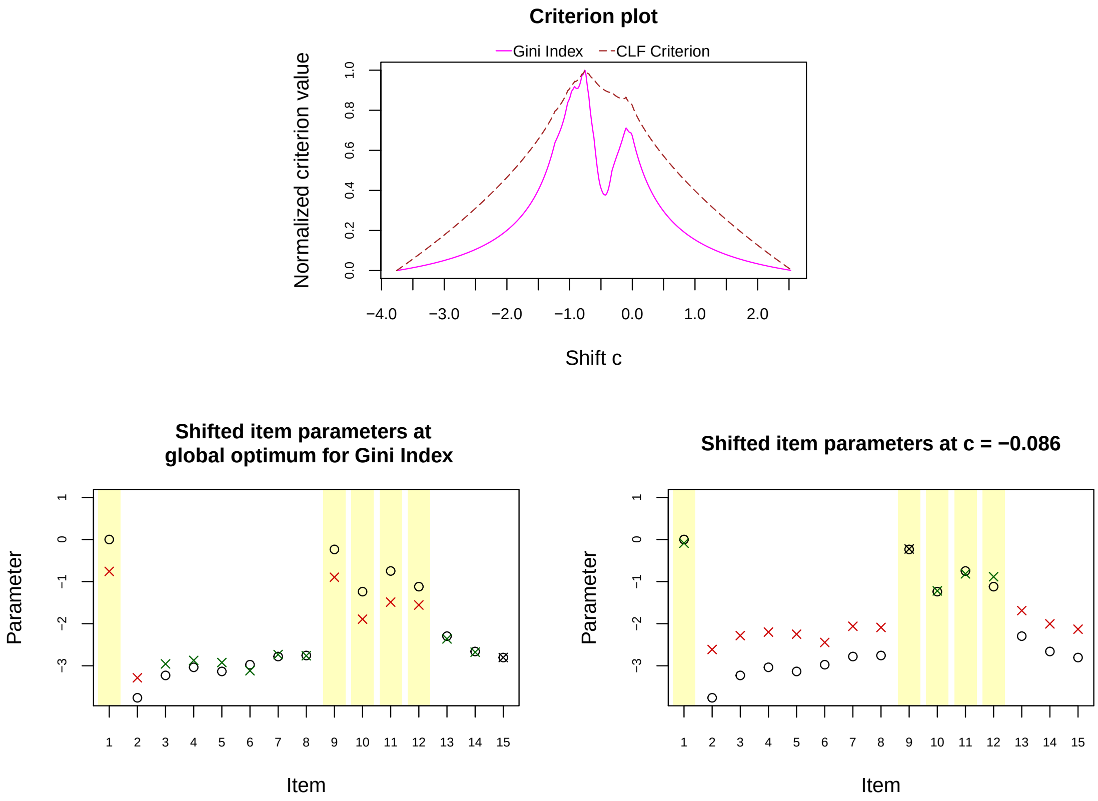

Figure 18: Criterion plot (top), shifted item parameters according to global (bottom left) and local optimum (bottom right), with items displaying statistically significant DIF indicated in red and items not displaying statistically significant DIF indicated in green, based on estimated item parameters for the first three factors of the international personality item pool. The item parameters symbolized by circles belong to the female group, those symbolized by crosses to the male group. Items indicated by yellow highlighting are referred to in the text.

systematically different distributions on these dimensions (cf. Roussos & Stout, 1996).

What we do find, however, is a less obvious pattern: Those items that show notable DIF in the same direction (harder to agree to for female respondents) in the globally optimal solution (Figure 18, bottom left) and interlock or are very close in the locally optimal solution (Figure 18, bottom right), are the inversely phrased items: items 1 (“A1: Am indifferent to the feelings of others”), 9 (“C4: Do things in a half-way manner.”), 10 (“C5: Waste my time.”), 11 (“E1: Don’t talk a lot.”) and 12 (“E2: Find it difficult to approach others.”). These items are indicated in Figure 18 (bottom) by yellow highlighting.[7] In this example, the inversely phrased items form a cluster corresponding to the second peak in the criterion plot, that is clearly distinguishable for the Gini Index. An interpretation of this cluster from the multidimensionality perspective of DIF is that these inversely phrased items form a method factor (c., e.g., Weijters, Baumgartner & Schillewaert, 2013) on which female and male respondents differ, possibly due to gender specific social desirability.

[7]Note that item 2 (“A2: Inquire about others’ well-being.”) also shows notable DIF in the globally optimal solution (Figure 18, bottom left), but this items is not part of the same cluster as the inversely phrased items because for item 2 the DIF goes in the other direction (easier to agree to for female respondents).

F Mathematical derivation of possible locations of optima

Let $a_j = \tilde{\beta}_j^{(g_1)} - \tilde{\beta}_j^{(g_2)}$ be the distance between the initial item parameter estimates in the two groups for item j, with $j = 1, \ldots, m$. The item-wise absolute distance on the shifted scale $d_j(c)$ used in the main text then corresponds to $d_j(c) = |a_j + c|$. For brevity, we will denote $d_j(c)$ as d_j.

Without loss of generality, we assume that the a_j are sorted in non-decreasing order and $a_m = 0$. We will in the following use s_j for the sign of $a_j + c$ and r_j for the rank of d_j in non-decreasing order. Note that s_j is the first derivative of d_j wrt. c.

F.1 Locations of optima for the Gini Index

In our simplified notation, the Gini Index can be written as as

$$\mathrm{GI}(c) = \frac{2 \cdot \sum_{j=1}^{m} r_j \cdot d_j}{m \cdot \sum_{j=1}^{m} d_j} - \frac{m+1}{m}.$$

This formula can be separated in a trivial part and an interesting part by writing $\mathrm{GI}(c) = \frac{2}{m} f(c) - \frac{m+1}{m}$ with $f(c)$ defined as

$$f(c) = \frac{\sum_{j=1}^{m} r_j d_j}{\sum_{j=1}^{m} d_j}$$

The numerator of the first derivative of f wrt. c is given by

$$num(f'(c)) = \left(\sum_{j=1}^{m} r_j s_j\right)\left(\sum_{k=1}^{m} d_k\right) - \left(\sum_{j=1}^{m} s_j\right)\left(\sum_{k=1}^{m} r_k d_k\right) \tag{1}$$

Resorting the summands by $s_j d_k$, this can be written as

$$num(f'(c)) = \sum_{j,k=1}^{m} (r_j - r_k) s_j d_k = \sum_{j,k=1}^{m} (r_j - r_k) s_j s_k (a_k + c).$$

Observe that this value is non-continuous at every position where either any sign s_j switches, or where the ranks of two neighboring values change, i.e., if $d_j = d_k$. Note that since all values start negative, the second condition can be written as $a_j + c = -c - a_k$ (regardless of the order of j and k). So all positions c where f' is not continuous are all points

$$c = -a_j \tag{2}$$

or

$$c = -\frac{a_j + a_k}{2}$$

for any a_j and a_k; these are $\frac{m(m+1)}{2}$ positions.

Between any two such positions, s_j and r_j are constant, f is linear in the denominator, and the denominator is always positive.

An important and non-trivial observation is that the numerator is constant with respect to c, since

$$\begin{aligned} num(f'(c)) &= \sum_{j,k=1}^{m} (r_j - r_k) s_j s_k (a_k + c) \\ &= \left(\sum_{j,k=1}^{m} a_k (r_j - r_k) s_j s_k\right) + c \left(\sum_{j,k=1}^{m} (r_j - r_k) s_j s_k\right) \\ &= \left(\sum_{j,k=1}^{m} a_k (r_j - r_k) s_j s_k\right) \end{aligned}$$

So all extrema of f are at the points where f' is not continuous. Note that the ranges $c < 0$ and $c > -a_1$ asymptotically approach zero and contain no extrema other than the minima at $\pm\infty$.

Further note that at any point where the rank switches, two neighboring ranks r_k and r_l are exchanged; assume $k < l$ wlog. At this position, s_k is still negative while s_l is positive. The second term of Equation 1 is continuous here as $d_k = d_l$, in the first term the sum of all d's is positive. As the rank r_l is increased by one while s_l is positive, and vice versa for k, $f'(c)$ steps to a higher value, potentially indicating a minimum, but no maximum.

So, the Gini index can only take a maximum at the points where $c = -a_j$ (Equation 2) and thus d_j is zero for any j. Since these are only m positions, we can easily find the maximal Gini index by testing all $c = a_j$ values and choosing the largest among them, in $O(m)$ steps.

F.2 Locations of optima for the CLF Criterion

In our simplified notation and for the two-group case with only one item parameter, i.e., for the Rasch model, the CLF Criterion[8] reduces to

$$\mathrm{CLF}(c) = \sum_{j=1}^{m} \sqrt{d_j}.$$

To compare with the Gini index, we consider the extrema of this index, too. The first and second derivatives are given by

$$\begin{aligned}\mathrm{CLF}'(c) &= \frac{1}{2}\sum_{j=1}^{m} s_j \frac{1}{\sqrt{d_j}} \\ \mathrm{CLF}''(c) &= -\frac{1}{4}\sum_{j=1}^{m} d_j^{-\frac{3}{2}}\end{aligned}$$

We observe that the derivatives are not defined at any point where $d_j = 0$. The second derivative is negative everywhere in between these points. So in these areas, we have no minima, and exactly one maximum. At the points where $d_j = 0$, the first derivative reaches a pole with a sign switch from negativ to positive, reflected by a local minimum (with a cusp) at the index itself. Consequently, all minima are at $c = a_j$, and the global minimum can be found most efficient by picking the smallest $\mathrm{CLF}(c)$ among those.

This means that for both criteria, we need to search only through the positions $a_1, \ldots, a_m$ to find all optima.

[8]For mathematical simplicity, we have not changed the sign of the CLF in this appendix. Therefore, the interesting optima for the CLF Criterion in the appendix are minima, while those for the Gini Index are maxima.

References

Ackerman, T. A. (1992). A didactic explanation of item bias, item impact, and item validity from a multidimensional perspective. *Journal of Educational Measurement*, *29*(1), 67–91.

Asparouhov, T. & Muthén, B. (2014). Multiple-group factor analysis alignment. *Structural Equation Modeling: A Multidisciplinary Journal*, *21*(4), 495–508.

Bechger, T. M. & Maris, G. (2015). A statistical test for Differential Item Pair Functioning. *Psychometrika*, *80*(2), 317–340.

Chalmers, R. P. (2012). `mirt`: A multidimensional Item Response Theory package for the R environment. *Journal of Statistical Software*, *48*(6), 1–29. `https://CRAN.R-project.org/package=mirt`.

Glas, C. A. W. & Verhelst, N. D. (1995). Testing the Rasch model. In G. H. Fischer & I. W. Molenaar (Eds.), *Rasch Models – Foundations, Recent Developments, and Applications* chapter 5. New York: Springer-Verlag.

Kopf, J., Zeileis, A., & Strobl, C. (2015a). Anchor selection strategies for DIF analysis: Review, assessment, and new approaches. *Educational and Psychological Measurement*, *75*(1), 22–56.

Kopf, J., Zeileis, A., & Strobl, C. (2015b). A framework for anchor methods and an iterative forward approach for DIF detection. *Applied Psychological Measurement*, *39*(2), 83–103.

Muthén, B. & Asparouhov, T. (2014). IRT studies of many groups: The alignment method. *Frontiers in Psychology*, *5*, 978.

Pohl, S., Stets, E., & Carstensen, C. (2017). Cluster-based anchor item identification and selection. Technical Report 68, Leibniz Institute for Educational Trajectories, National Educational Panel Study, Bamberg.

Rasch, G. (1960). *Probabilistic Models for Some Intelligence and Attainment Tests.* Chicago, London: The University of Chicago Press.

Reckase, M. (2009). *Multidimensional Item Response Theory.* New York: Springer.

Revelle, W. (2018). ***psych**: Procedures for Psychological, Psychometric, and Personality Research.* `https://CRAN.R-project.org/package=psych`.

Roussos, L. & Stout, W. (1996). A multidimensionality-based DIF analysis paradigm. *Applied Psychological Measurement*, *20*(4), 355–371.

Trepte, S. & Verbeet, M. (Eds.). (2010). *Allgemeinbildung in Deutschland – Erkenntnisse Aus Dem SPIEGEL Studentenpisa-Test.* Wiesbaden: VS Verlag.

Wang, W.-C., Shih, C.-L., & Sun, G.-W. (2012). The DIF-free-then-DIF strategy for the assessment of Differential Item Functioning. *Educational and Psychological Measurement*, *72*(4), 687–708.

Weijters, B., Baumgartner, H., & Schillewaert, N. (2013). Reversed item bias: An integrative model. *Psychological Methods*, *18*.

Woods, C. M. (2009). Empirical selection of anchors for tests of Differential Item Functioning. *Applied Psychological Measurement*, *33*(1), 42–57.

Wright, B. D. & Stone, M. (1999). *Measurement Essentials*. Wilmington: Wide Range Inc.

Zeileis, A., Strobl, C., Wickelmaier, F., Komboz, B., & Kopf, J. (2018). *psychotree: Recursive Partitioning Based on Psychometric Models*. https://CRAN.R-project.org/package=psychotree.
